# The effect of differential mineral shrinkage on crack formation and network geometry

**DOI:** 10.1038/s41598-022-23789-3

**Published:** 2022-12-23

**Authors:** Jeremy E. Trageser, Chven A. Mitchell, Reese E. Jones, Edward N. Matteo, Jessica M. Rimsza, Laura J. Pyrak-Nolte

**Affiliations:** 1 Center for Computing Research, Sandia National Laboratories, Albuquerque, NM USA; 2 Department of Earth, Atmospheric, and Planetary Sciences, Purdue University, West Lafayette, IN USA; 3 Nuclear Waste Disposal Research and Analysis, Sandia National Laboratories, Albuquerque, NM USA; 4 Mechanics of Materials, Sandia National Laboratories, Livermore, CA USA; 5 Geochemistry Department, Sandia National Laboratories, Albuquerque, NM USA; 6 Department of Physics and Astronomy, Purdue University, West Lafayette, IN USA; 7 Lyles School of Civil Engineering, Purdue University, West Lafayette, IN USA

**Keywords:** Civil engineering, Geophysics, Structural materials

## Abstract

Rock, concrete, and other engineered materials are often composed of several minerals that change volumetrically in response to variations in the moisture content of the local environment. Such differential shrinkage is caused by varying shrinkage rates between mineral compositions during dehydration. Using both 3D X-ray imaging of geo-architected samples and peridynamic (PD) numerical simulations, we show that the spatial distribution of the clay affects the crack network geometry with distributed clay particles yielding the most complex crack networks and percent damage (99.56%), along with a 60% reduction in material strength. We also demonstrate that crack formation, growth, coalescence, and distribution during dehydration, are controlled by the differential shrinkage rates between a highly shrinkable clay and a homogeneous mortar matrix. Sensitivity tests performed with the PD models show a clay shrinkage parameter of 0.4 yields considerable damage, and reductions in the parameter can result in a significant reduction in fracturing and an increase in material strength. Additionally, isolated clay inclusions induced localized fracturing predominantly due to debonding between the clay and matrix. These insights indicate differential shrinkage is a source of potential failure in natural and engineered barriers used to sequester anthropogenic waste.

## Introduction

A challenge in using natural and engineered materials as barriers for subsurface sequestration of anthropogenic waste (e.g., nuclear waste) is that these materials evolve over time in response to physical and chemical processes. A critical component of this evolution is the initiation, propagation, and coalescence of cracks that causes mechanical instabilities and potentially introduces highly conductive flow paths. While methods exist to predict the formation and propagation of cracks in brittle materials^1–3^, models are needed that account for heterogeneous materials composed of minerals with varying strengths, interfacial bonding conditions, and responses to chemical stimuli. Differential shrinkage is of particular concern in polymineralic materials used as barriers. Volumetric mineral shrinkage can arise from changes in humidity when fluids are injected or withdrawn from a subsurface site, or from changes in temperature that occur during nuclear waste disposal. A drying front induced by changing conditions can cause localized differential shrinkage that results in induced deformation gradients and non-uniform stress fields. Failure of a polymineralic material from dehydration can occur if the induced tensile stresses are greater than the mineral strength, which causes the mineral to crack, or if a mineral shrinks at a rate faster than other components, which causes debonding at the interface between constituent materials. Cracking of a polymineralic material during dehydration will vary with the relative strengths of the interfaces and the constituent material. A key question is whether the induced cracks will exist in isolation or propagate and coalesce into a network that diminishes the hydraulic integrity of the material by forming connected flow paths.

Here, we demonstrate that numerical simulations can simulate crack networks induced from the differential shrinkage of minerals during drying, and that the induced crack networks are analogous to those observed from laboratory tests on geo-architected rock samples. The use of geo-architected samples enabled repeatable testing and control over the amount and spatial distribution of clay in a cement matrix. The most critical parameters for the formation of crack networks were the relative shrinkage rate of the individual materials, which was introduced by modifying the stress-free reference volumes, and the duration of drying. Locally varying crack patterns in clay-cement composites are not controlled by the interfacial bonding between the materials, but instead by their relative shrinkage rates. This work demonstrates that peridynamics (PD) simulations^4,5^ can be used to efficiently investigate and predict damage and fracturing in polymineralic composites that contain constituents with dissimilar dehydration properties. The findings suggest detailed mineralogical studies combined with numerical simulation can be used to predict the long-term caprock integrity of subsurface sequestration reservoirs and the structural integrity of natural or engineered barriers in nuclear waste storage where moisture contents change throughout the site’s life-cycle.

## Results

### Geo-architected rock

**Figure 1:**
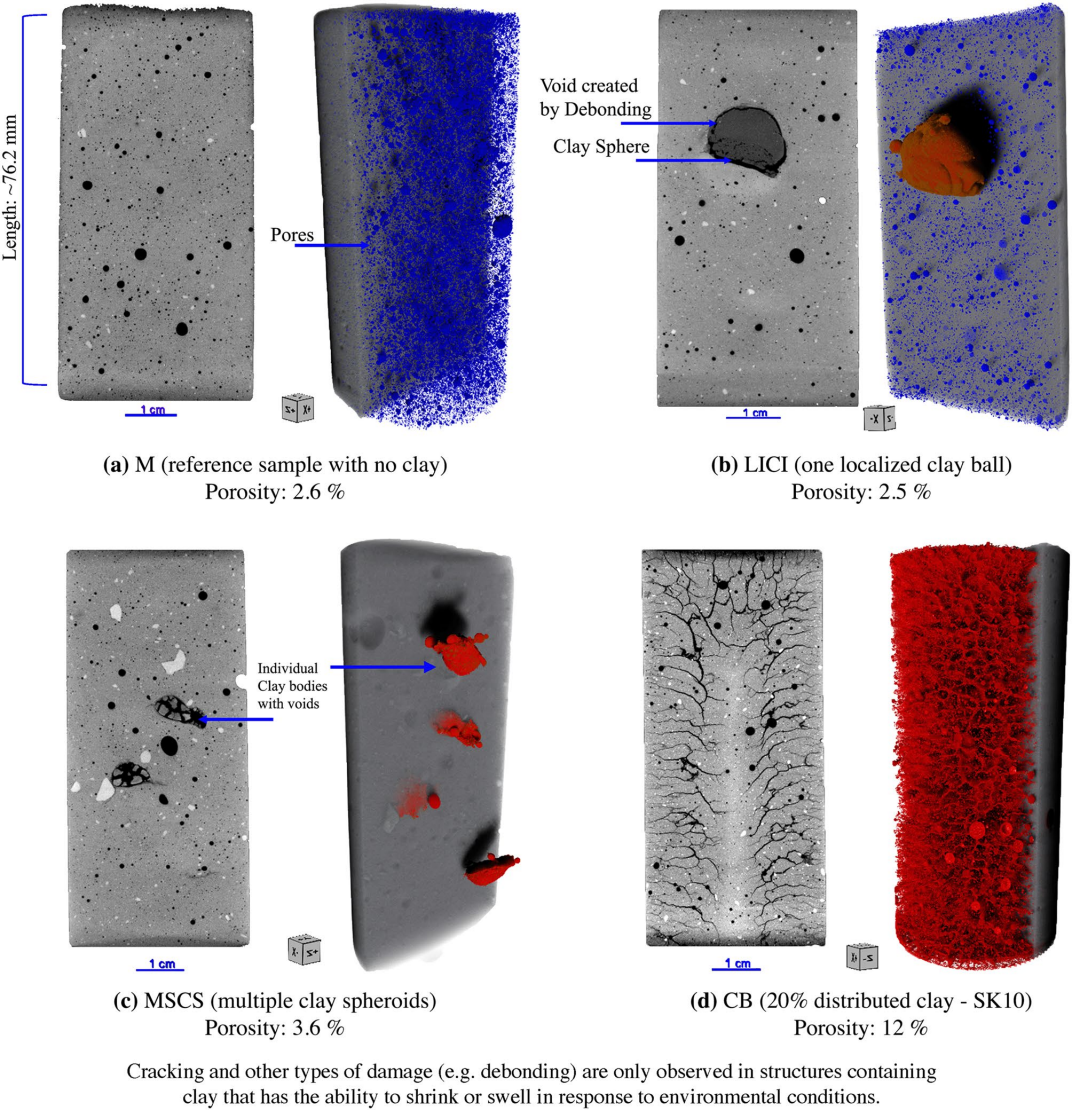
2D images from the center of the sample along the vertical axis, and 3D visualizations of the interior for the geo-architected samples after 6 full days of dehydration. The pixel edge length resolution is $\sim 40\; \upmu \text{m}$ and the average radius is 19.05 mm. All samples contain non-connected pores (displayed in blue) which are shown only for the reference sample (**a**) and the LICI sample (**b**). Clay inclusions are displayed in brown. All cracks are displayed in red, as shown for the MSCS sample (**c**) and the CB-SK10 sample (**d**). Cracks are only observed in samples containing localized or distributed clay which can result in a 40% reduction in material strength (a comparison between the reference and the CB-SK10 sample). Data are visualized with Dragonfly Pro software, Version 2020.2 for [Windows] from ORS^13^.

Geo-architected rocks were used to examine the formation of cracks from differential mineral shrinkage. A geo-architected rock is a rock analogue^6,7^ that is fabricated in the laboratory with natural constituents to develop controlled features and mineralogy for repeatable testing of hypotheses. Here, cylindrical samples (76.2 mm in length and 38.1 mm in diameter) with different clay distributions embedded in mortar were made with Ottawa sand^8^, ordinary Portland cement (OPC)^9^, and pre-soaked montmorillonite K10^10^, a commercially available expandable clay. The internal structure of the specimens included (i) a single large isolated clay inclusion (LICI) that occupied 11% of the sample volume, (ii) multiple small clay spheroids (MSCS) that occupied approximately 6% of the sample volume, and (iii) clay particles distributed throughout the bulk matrix (CB) (Fig. 1b–d). Reference samples composed only of mortar (M) (Fig. 1a) were also fabricated with a water-to-cement ratio of 0.87. This high water/cement ratio was intentionally used to obtain a reference sample with reduced intermediate strength and comparable water contents to that of the geo-architected rocks.

The CB synthetic rocks are characterized by a mixture that is rich in CaCo_3_, SiO_2_, and clay, a composition that is analogous to that of argillaceous rocks such as marls that are also clay-bearing carbonate rich porous media. The CB rock samples are also comparable to cement stabilized Marl, clayey soils, or clays which are used in several applications (e.g., embankments, tunnels, mining, engineered barriers, etc.). Marls are rocks that are comprised of 35% to 65% carbonate with the remaining percentage consisting of various quantities of clay and silt-sized particles^11^. The strength of clay-bearing rocks like marl vary with moisture content and mineral composition^12^ including the type of clay that is contained within the microstructure. Samples were also fabricated with different clay types to investigate the relation between chemical composition and moisture loss and the effect on induced crack formation for materials with different shrinkage rates. The CB sample group also includes samples with (i) different Montmorillonite compositions CB-SWY (Fig. S7d) and CB-STX (Fig. S7c) , (ii) a lower percentage of Montmorillonite CB-SK10-5 (Fig. S7e) , and (iii) a non-swelling clay CB-SKAO (Fig. S4) for which no shrinkage was expected. The LICI, MSCS, and CB-SK10 samples were cured for 7 days via submerged curing, while the CB-SWY, CB-STX, CB-SKAO, and CB-SK10-5 samples were cured for 14 days to improve material strength. Additional details regarding sample fabrication can be found in Supplementary Note 1 and Supplementary Note 2.

Material property testing of the geo-architected samples determined that the amount, location, and type of clay affected both the unconfined compressive strength (UCS) and the rate of moisture loss from the sample. Embedding swelling clay into the cement matrix tended to reduce the strength of a sample. During desiccation the swelling clay will tend to shrink so that it no longer provides load-bearing capacity to the pore spaces of the matrix. The CB samples, CB-SWY (1.15 MPa) and CB-SK10 (4.6 MPa), exhibited the lowest average UCS values, while the UCS for CB-STX (6.36 MPa) and CB-SK10-5 (8.05 MPa) were lower than the mortar reference sample (11.4 MPa) and the MSCS samples (11.05 MPa). The LICI sample had an intermediate average UCS value of 6.1 MPa, and the average UCS for the sample fabricated with non-swelling clay (CB-SKAO) was 7.7 MPa. Though the unconnected porosity of the samples were similar, the moisture loss in the distributed CB-SWY (38%) and CB-SK10 sample (21%) was 2.92 and 1.6 times higher than the observed moisture loss for the reference mortar sample (13%). In the initial sample group (reference versus LICI, MSCS, and CB-SK10) the greatest moisture loss occurred in the sample CB-SK10 which was observed, experimentally, to contained the most complex crack network after drying (Fig. 1d). A representative CB-SWY sample experienced the most moisture loss (1.8 times higher than CB-SK10), and the crack network that developed was dissimilar to that of CB-SK10 sample (Fig. 9d). Additional details on material properties can be found in Supplementary Note 4.

### Crack network topology

The nucleation and propagation of cracks in a polymineralic material that arise from differential shrinkage depends on the mineral strengths, the interfacial bonding strengths among the mineral constituents, and how the constituents are distributed throughout a sample. 3D X-ray microscopy (Zeiss Xradia 510 Versa) was used to image the samples immediately after removal from the curing tank and intermittently over a period of 6 days. The reference sample M, along with the CB samples (STX, SWY, SKAO, and SK10-5 ) were only scanned after removal from the curing environment, and then again after 6 full days of drying. The samples were dried inside the X-ray system at ambient relative humidity and a temperature of 28 °C that was maintained by the X-ray system.

**Figure 2:**
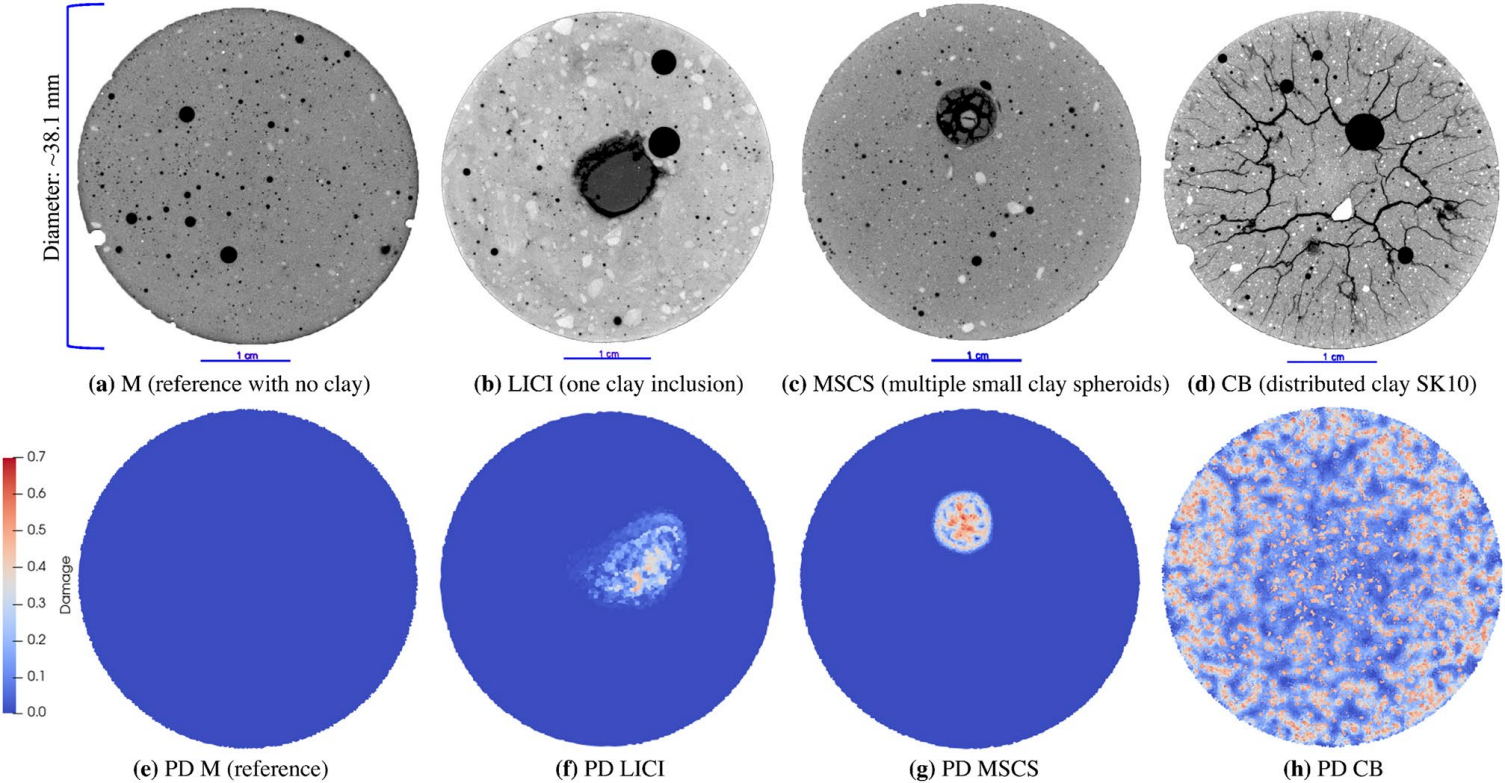
A comparison of the experimental and simulated damage, where the top row is comprised of two-dimensional (2D) images at the center of each geo-architected sample: (**a**) the reference sample with no visible cracks, (**b**) LICI and (**c**) MSCS with fractured regions in and surrounding the inclusions, and (**d**) the CB sample with many visible cracks. The bottom row is comprised of 2D images of the PD model results for each experimental structure: (**e**) is the data obtained for the reference model, (**f**) is data for the LICI sample, (**g**) is data for the MSCS sample, and (**h**) is data for the CB-SK10 sample. Damage is observed in both the experimental and PD models, and is localized and/ or distributed in areas that contain highly-shrinkable clay. All images are viewed along the transverse plane after six (6) full days of dehydration. Experimental data are processed with Dragonfly Pro software, Version 2020.2 for [Windows] from ORS^13^.

Two dimensional (2D) images and 3D X-ray reconstructions of samples LICI, MSCS, CB-SK10 and M after 6 days of drying show that the amount and location of cracking differs among the samples (Fig. 1). For the CB sample group, the amount and location of cracking is similar for CB-SK10 and the CB-STX samples (Fig. 9b,c) but differs in comparison to the CB-SWY and CB-SK10-5 synthetic rocks (Fig. 9d,e). When clay inclusions are isolated in distinct shapes (e.g., samples LICI and MSCS), cracking is confined to the inclusions and to debonding between the inclusions and the mortar (Figs. 1b,c and 2b,c). Debonding is the process whereby the clay-cement interface separates due to the rupturing of bonds which leads to the formation of a crack. The consequence of this phenomena is particularly evident in the case of the LICI synthetic rock where a discontinuity exists in weaker areas where debonding at the interface and along the circumference of the localized body of clay occurred. Conversely, when swelling clay particles are distributed throughout the matrix as in sample CB-SK10 (Figs. 1d and 2d) or CB-STX (Fig. 9c), complex crack networks are formed that appear to initiate from the edge of the sample and then propagate inward, essentially following the propagation direction of the drying front. The M (reference) sample exhibited no cracks or damage from drying (Figs. 1a and 2a). Additionally sample CB-SKAO (the sample with 20% distributed calcined Kaolinite) also exhibited no cracks or damage from drying (Fig. S4).

**Figure 3:**
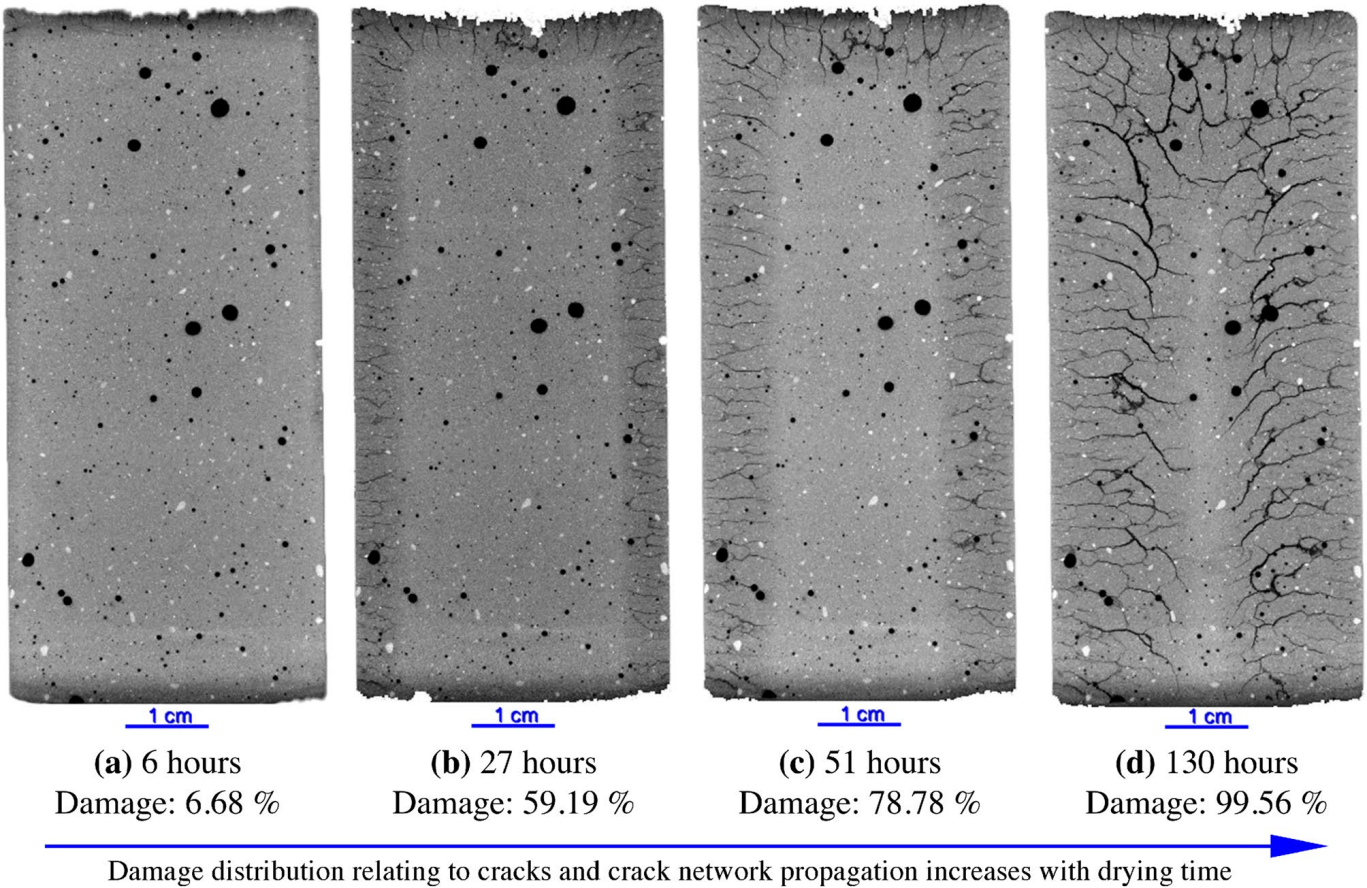
2D images from the center of the interior of the CB-SK10 geo-architected sample with 20% montmorillonite showing time dependent deformation during moisture loss, (**a**–**d**) presents drying front progression, reduction of the coherent undamaged material and growth of the crack network. Data are visualized with Dragonfly Pro software, Version 2020.2 for [Windows] from ORS^13^.

**Figure 4:**
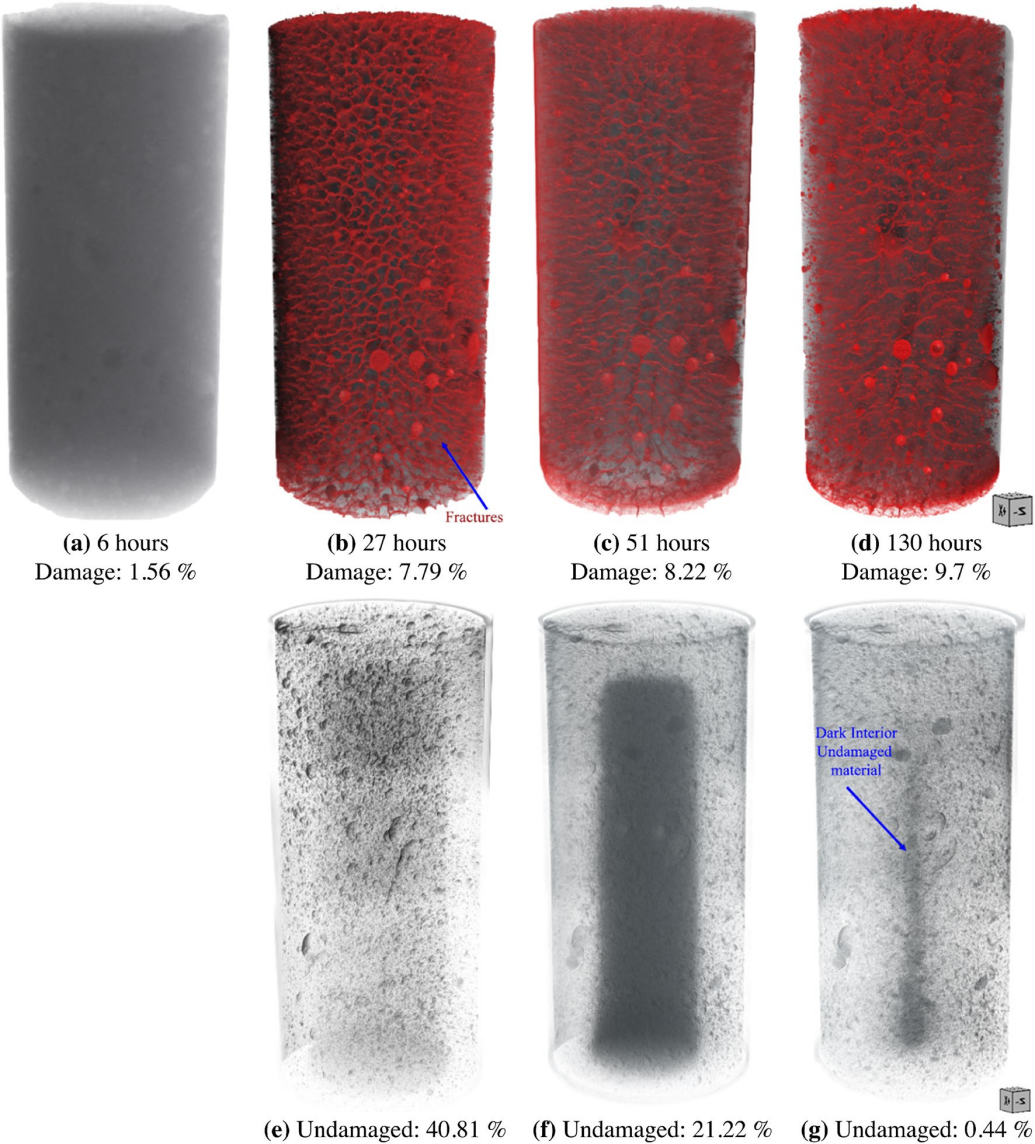
3D visualizations of the interior and drying shrinkage deformation of a specimen with 20% montmorillonite distributed within the framework during the experimental dehydration period, where (**a**–**d**) shows the growth of the crack network in 3D, and (**e**,**f**) shows the reduction of the undamaged material zone (shrinking dark columns) and evolution of the drying front. The undamaged data for the initial sample is not shown as  93% of the sample remains undamaged. Data are visualized with Dragonfly Pro software, Version 2020.2 for [Windows] from ORS^13^.

Time-lapsed 3D X-ray tomography was performed on a CB-SK10 sample to determine the relationship between the extent and location of the cracks relative to the drying front. Figure 3 shows 2D cross-sections from the center of a CB-SK10 sample after 6, 27, 57 and 130 h of drying. After 6 h of drying under ambient conditions, the drying front, which occupies 6.7% volume of the CB-SK10 sample, is observed to extend 1–2 mm into the right edge, top and bottom of the sample with no apparent cracks for a voxel resolution of $40 \; \upmu \text{m}$ (Fig. 3a). As time progressed, the drying front moved into the sample and a crack network is observed in the dry portions of the sample (Fig. 3b–d). The cracks propagated from the edge of the sample (Fig. 4b) and terminated on the drying front. An increase in aperture with extended drying is also observed (Fig. 4c), and the final value for $N_c$ which is the number of cracks extracted from Fig. 4d (52% of which are intersecting) was 197 (Table S5).

Though the LICI and MSCS samples did not undergo observable in-matrix cracking, the inclusions in LICI experienced a 26% decrease in volume that resulted in 0.7% increase in crack volume within and surrounding the clay body. The clay inclusions in the MSCS sample (see Figs. 1c and 2c) experienced a cumulative decrease in volume of 16.5% that also resulted in a 0.7% increase in crack volume from interfacial debonding and cracking that occurred in and around the clay. The total porosity of the CB-SK10 sample increased from 3.75 to 12.6% because of the formation of an intricate crack network (Figs. 3d and 4d).

**Figure 5:**
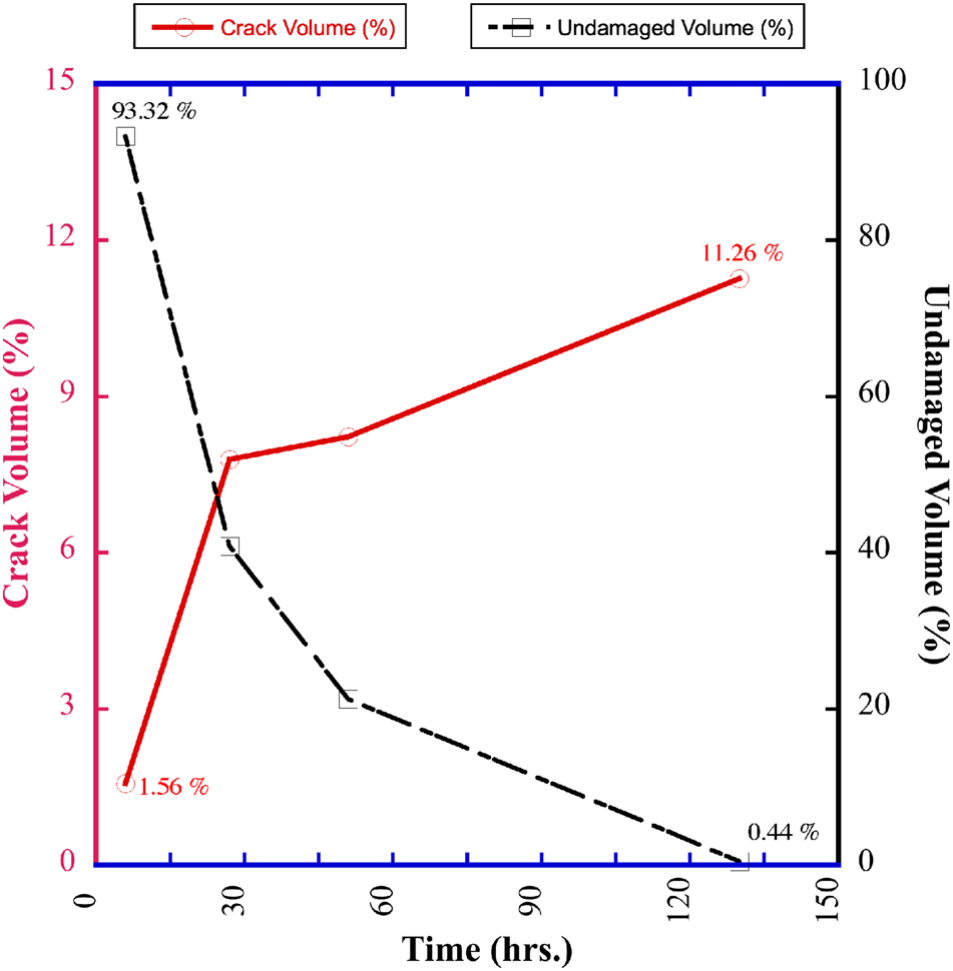
The total crack percent volume (left y-axis) and percent volume of the undamaged zones (right y-axis) in a CB-SK10 geo-architected sample that was monitored for > 130 h of moisture loss with a Zeiss Xradia 3D X-ray Microscope at ambient relative humidity and an average system temperature of 28 °C. The data obtained show that an increase in the volume and distribution of cracks naturally results in a reduction of the zones within the structure where no damage has occurred. Here the solid line relates to the left y-axis and the dashed line is associated with the right y-axis.

The evolution of damage as a function of drying time for sample CB-SK10 is shown in Figs. 3a and 4d. Image subtraction was used to enhance the visualization of the undamaged regions (Fig. 4e–g) and quantify the volume of intact material untouched by the advancing crack network as the CB-SK10 sample dried. This was achieved by subtracting the current volume data of a 3D snapshot of the CB-SK10 sample from the initial condition visualized in Fig. 4a. The magnitude of the cracked versus undamaged volumes as a function of time are shown in Fig. 5. As the network evolves (Fig. 4a–d), the percent volume of the damaged regions in the matrix of sample CB-SK10 increased from 4.44% at the end of the first 3D X-ray scan (6 h of drying in Fig. 3a) to 99.56% at the end of the drying period (Fig. 3d).

Overall, the samples containing montmorillonite clay exhibited multiple cracks, many of which coalesced at the end of the drying period. Moisture loss induced different crack types: from intricate crack networks (Fig. 1d), to debonding cracks at the mortar-clay interface (Fig. 1b), to cracks where wisps of clay migrated out of the localized zones (observed in Fig. 1c), and cracks that formed internally in individual clay inclusions (Fig. 2b,c). The “crack wisps” are hypothesized to arise from clay transport during vibration at the point of molding—see Supplementary Note 2).

### Simulated damage

A key question raised by the data and images from the experiments on the geo-architected samples is: Is clay shrinkage alone sufficient to induce the intricate crack network observed in the CB samples or do other material properties contribute to the final crack patterns? Numerical simulations were used to address this question since modeling can test a range of material properties that is not possible in the laboratory. In general, simulating damage nucleation and propagation is challenging, particularly for materials with heterogeneities, such as clay inclusions in a mortar matrix. Here a novel peridynamics, PD, model was used to simulate, drying with a nonlocal diffusion process and the cracks that result from the predicted damage field. With this model several characteristics of the physical experiments were successfully reproduced, allowing for numerical evaluation of the impact of critical material properties on fracture networks. The simulations used the experimental sample geometries for the clay-mortar systems (M, LICI, MSCS) and for the CB-SK10 sample 20% of the sample geometry is clay consistent with the experimental systems. The model geometries are illustrated in Figs. S1 and 6.

In the simulations, the first assumption was that most of the water loss and shrinkage occurs in the clay portion of the models. Second, the elastic and fracture properties of the OPC were assumed to remain unchanged during the drying process because negligible shrinkage of the mortar (M) was observed in the experiments. The assumed properties for the mortar matrix include: a fracture toughness of ($K_{IC}$) = 2.8 $\text {MPa}\sqrt{\text {m}}$, a shear modulus of $G =12.0$ GPa, and a bulk modulus of $K = 20.0$ GPa^14^. The clay properties were consistent with published values for Montmorillonite, the same clay used in the experiments. At room temperatures, dehydration is from water trapped between the clay sheets^15^, causing the d-spacing of the basal plane to decrease from 15 to 12 Å, consistent with a decrease from two to one layer of water between the basal planes^16^. Clay dehydration also strengthens the clay due to the increase in hydrogen bonds to stabilize the material, with increases in elastic moduli by 15–20%^16^. Therefore, in this study initial pre-dehydration moduli values of K = 12.7 GPa and G = 6.6 GPa for Montmorillonite were based on previously published results^17^ with an assumed increase to K = 14.6 GPa and G = 7.6 GPa following dehydration. In additional to mechanical properties of the clay, the fracture toughness $K_{IC}$ is needed to calculate the critical stretch parameter $s_c$ (see Eq. (5)) that dictates the material strength in the PD model. See Supplementary Note 12 and Note 13 for more information on the PD model. An initial $K_{IC}$ value of 0.08 $\text {MPa}\sqrt{\text {m}}$ is used for the hydrated clay based on average reported values in literature for clay soil^18^, kaolinite^19^, and Iro and Ewuya clay^20^. $K_{IC}$ values were reported to nearly double during dehydration^18^. Therefore, a $K_{IC}$ value of 0.18 $\text {MPa}\sqrt{\text {m}}$ was used for the dehydrated clay. The fracture and mechanical properties for the OPC and clay (hydrated and dehydrated) used in this study are included in Table S8.

**Figure 6:**
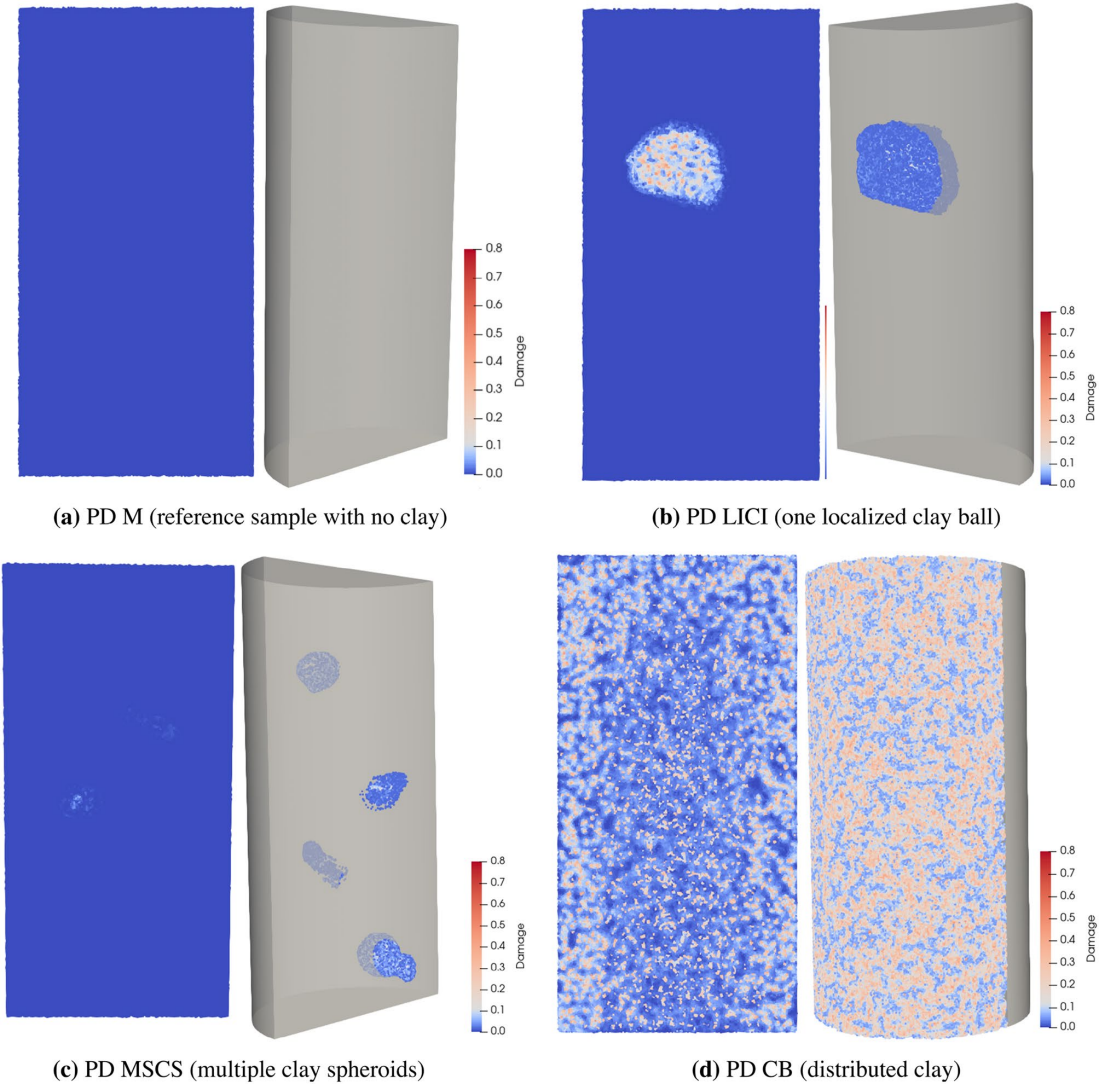
2D image cross-sections from the center of the results of the PD simulations along the vertical axis and 3D visualizations of the interior. Damage is displayed above the threshold of 0.01 in the 3D images which emphasizes that damage is localized around the clay inclusions. The PD model results adequately reflect the experimental observations shown in Fig. 1.

**Figure 7:**
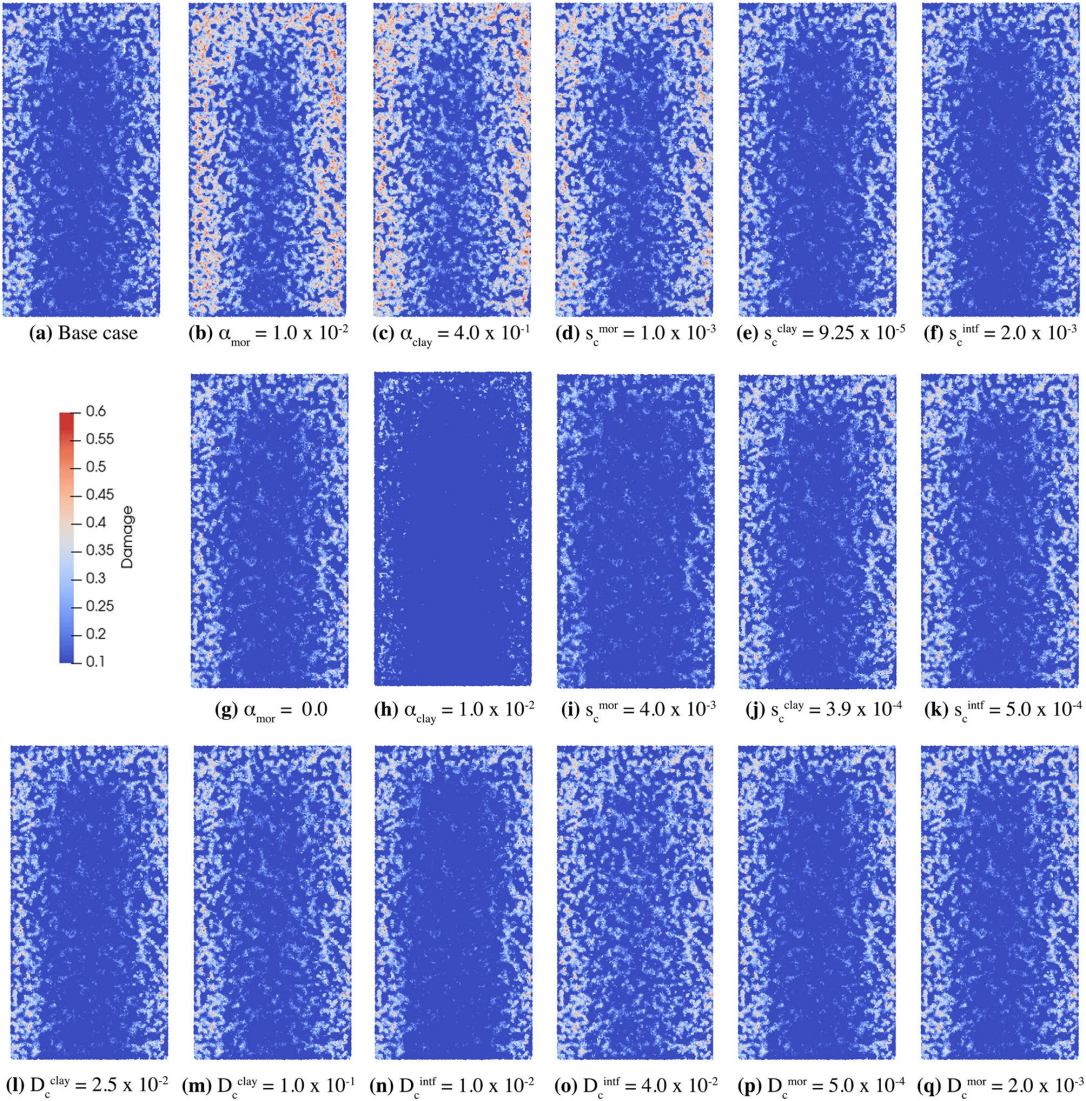
The final crack patterns among mortar nodes for the CB structure parameter study, viewed as a cross section of the model structure. The base case, (**a**), corresponds to parameters shown in Table S8 and “Methods” section: “Boundary conditions”. The remaining images correspond to the same parameters with a single parameter modified as specified in the associated caption. The parameters $\alpha$, $s_c$, and $D_c$ describe the shrinkage, critical stretch, and diffusivity and their corresponding superscripts/subscripts denote the material: mortar (mor), clay, or interface (intf). The most significant changes in the distribution of damage were observed when altering the shrinkage $\alpha$ (particularly for clay) and the critical stretch parameter $s_c$. The diffusivity, was observed to have little impact on the damage distribution.

The experimental results (see Figs. 1a and 2a and numerical results (see Figs. 2e and 6a) for sample M, which contained no clay, are both characterized by a lack of fracturing. The numerical results for the localized clay inclusions samples (LICI and MSCS) are characterized by cracks localized within and around the clay inclusions (see Fig. 2b,c,f,g). These numerical experiments compare well with the LICI experimental results observed in Figs. 1b and 2b, and the MSCS experimental results seen in Figs. 1c and 2c. In order to study sample CB, a parameter study was conducted to determine the sensitivity of the fracture patterns to changes in the material properties of the clay and mortar. The parameter study focused on the CB geometry since it exhibited the most complex crack patterns. By varying a single parameter from a set of base case parameters, the impact of each parameter on the fracture pattern was explored. The numerical models included eight different parameters that were varied: the water diffusivity and critical stretch of the mortar, clay, and interfaces as well as the shrinkage of the clay and mortar. The diffusivity, critical stretch, and shrinkage were all material parameters that varied the most between the two materials (the clay and the cement matrix) and during hydration, and were therefore expected to have the most significant effect on the resulting crack networks. Images from the PD models are presented in Fig. 7.

**Figure 8:**
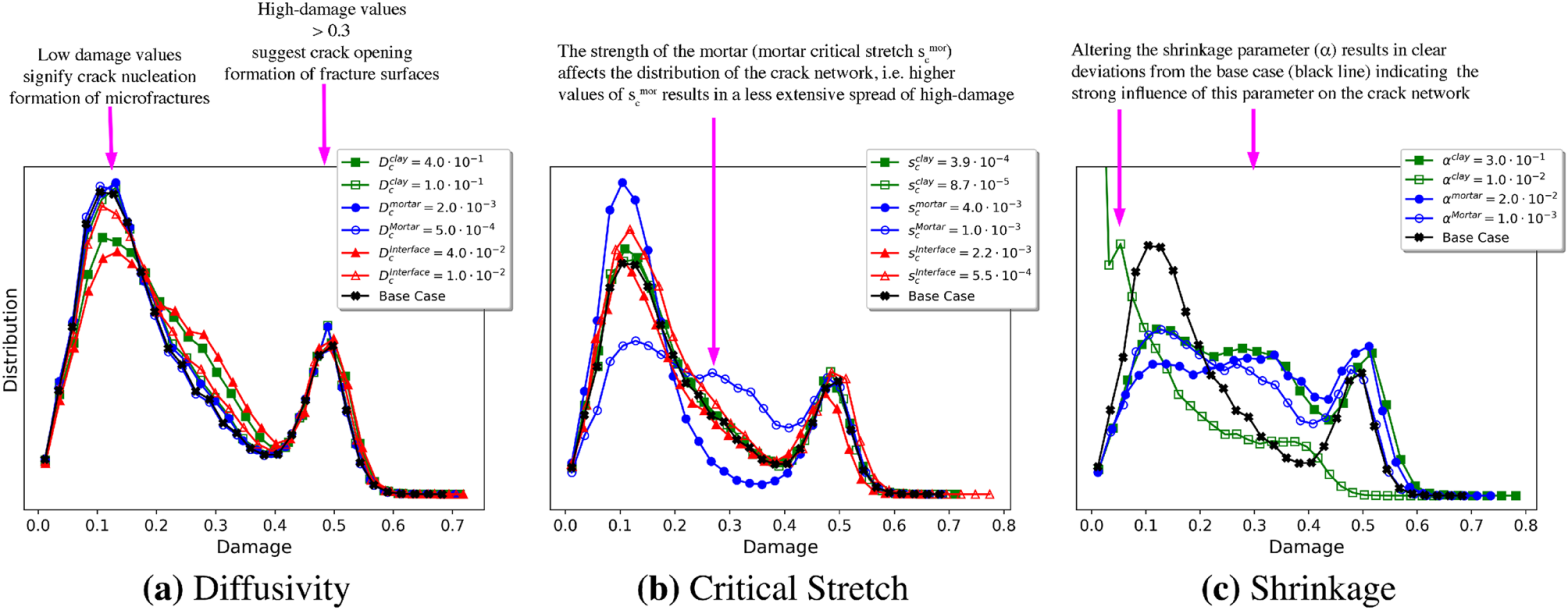
Histograms describing the distribution of damage among nodes in the numerical experiments after six (6) full days of dehydration. The bucket values on the x-axes are damage (see Equation (12) in Supplementary Note 12), which describes the number of broken bonds at a node. For example, 0.0 corresponds to no broken bonds and 0.6 corresponds to $60\%$ of bonds at a node being broken. The y-axis provides the quantity of nodes which fall into each bucket. Many of the plots have two peaks, the first of which occurs for damage near 0.15 which likely corresponds to crack nucleation and the formation of microfractures; the second peak with damage near 0.5 is primarily from the formation of fracture surfaces, particularly due to debonding between the clay and mortar. From the histograms, it is clear the most sensitive parameters are the shrinkage of both the clay and mortar and the critical stretch of the mortar.

Damage to a node which discretizes the material (see Equation (12) in Supplementary Note 12) occurs when a PD bond breaks, resulting in a loss of connectivity between the corresponding nodes that make up the bond. The damage at the node is the proportion of broken bonds connected to the node compared to the original number of bonds connected to the node. For example, if 10% of bonds connected to a node are broken then the damage would be 0.1. Histograms of the distribution of node damage during drying for each parameter perturbation are presented in Fig. 8.

In Fig. 8, two primary peaks are observed in the damage histograms, one at 0.15 (low) damage and the other at 0.5 (high) damage. Nodes with lower damage values (0.05–0.3) frequently signify crack nucleation, while higher damage values (0.3+) suggest the formation of surfaces and open cracks. Deviation from the base case identifies that changes in the crack pattern occurred when the material properties are varied. By comparing Fig. 8c with Fig. 8a,b it is clear the shrinkage parameter for clay $\alpha ^{\text {clay}}$ and the shrinkage parameter for mortar $\alpha ^{\text {mortar}}$ (see (6)) exhibited the strongest influence on the extent of the crack network. Decreasing the amount of clay shrinkage from $\alpha = 4.0 \times 10^{-1}$ to $1.0 \times 10^{-2}$ (see Figs. 7c,h, and 8c) resulted in a higher concentration of lightly damaged nodes, consistent with breaking 5–15% of the bonds between material nodes. This indicates microfracturing, but minimal overall loss of strength. For larger values of clay shrinkage (e.g., $\alpha = 4 \times 10^{-1}$ in Fig. 7c), more debonding occurs and nodes with 20–40% damage appear. This is consistent with the formation of fracture surfaces. Moreover, the strength of the mortar, which is described by the mortar critical stretch $s_c^{\text {mor}}$ in the PD model, significantly altered the distribution of damage in the system (see Fig. 8b). Specifically, larger mortar critical stretch values result in less extensive damage, as seen in Fig. 7i. The smaller values and lower concentration of damage indicate minor deterioration within the material from microcracks. Alternatively, smaller mortar critical stretch values resulted in more extensive damage, which is exemplified in Fig. 7d. The large damage values and higher concentration of damage are consistent with the formation of fracture surfaces. Interestingly, Fig. 8b demonstrates that the damage and resulting crack network in the CB systems were not particularly sensitive to the strength of clay (see Fig. 7e,j) or the strength of the clay-mortar interface (see Fig. 7f,k), with only a marginal effect when compared with many other parameter perturbations in Fig. 7. Generally, the clay and the clay-mortar interface fractures earlier and faster than mortar, so that changes to the low strength clay and clay-mortar interface (defined by the critical stretch parameter (5) in the PD model) have limited effect on the damage that results from fracturing in these systems.

### Differential moisture loss

Differential shrinkage was identified by the PD simulations as the dominant mechanism driving the fracturing observed in these polymineralic materials. Differential shrinkage of the constituent clay arises as moisture is lost from the microstructure. Thermogravimetric analysis with differential scanning calorimetry (TGA-DSC) was used to understand the moisture content of the materials. TGA-DSC provided insights on the rate of change of mass and native moisture content which relates to the material dehydration, and revealed clear differences in mass loss (moisture loss) between the swelling and non-swelling materials. Clear differences are observed, and the OPC and calcined Kaolinite (Fig. S2d,e) behave differently in comparison to the Montmorillonites. The initial moisture loss estimated from the mass loss at the first peak was 0.74% for OPC (130 °C), 0.35% for Kaolinite (66 °C), 5.18% for K10 (110 °C), 7.21% STx-1b (126 °C), and SWy-3 7.04% for SWy-3 (117 °C) (Fig. S2a–e). Initial mass loss is attributed to the loss of physically bound water. Interestingly up to 100 °C the Montmorillonite clay dehydration rates follow the same path with a gradient of 0.0005%/°C, and water weight loss of 4.9% for SWy-3 and STx-1b, and 4.4% for K10. This is an indication that for all types of Montmorillonite investigated here the rate of dehydration is fairly similar up to 100°. For a heat flow rate of 20 °C/min, nearly the same percent of water is lost and any shrinkage due to water loss for these material is comparable. Conversely due to its minimal water loss, the rate of differential shrinkage is expected to be negligible in samples containing the calcined Kaolinite. All materials experienced a total mass loss of 1.5% in the calcined kaolinite, 15.6% in STx-1b, 13.5% in SWy-3, 11.9% in K10, and 5.3% in OPC. Such differences translate into variations in material dehydration and shrinkage rates. The mass loss versus heat flow curves and the mass lost per unit of material can be observed in Figs. S2 and S3.

### Crack network comparison

**Figure 9:**
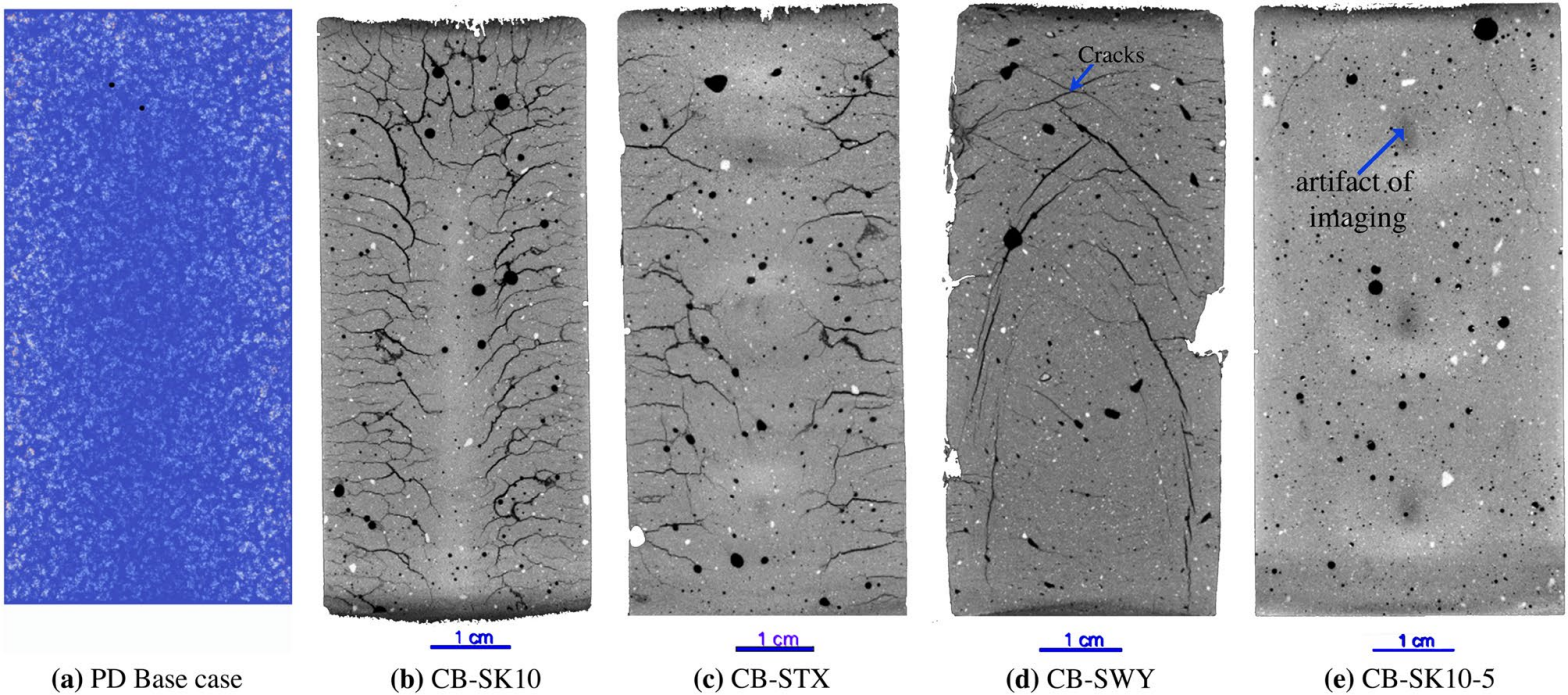
2D side view images taken from the center of the Base Case of the (**a**) simulated and experimental (**b**) CB-SK10, (**c**) CB-STX, (**d**) CB-SWY, and (**e**) CB-SK10-5 data. Here the observed crack networks are different for the different clay materials, and a greater distribution of damage is observed for the samples fabricated with “some type” of Montmorillonite. These images are later binarized for further analysis and comparison between the data.

To explore the effect of materials with different shrinkage rates, the CB sample group included geo-architect samples that contained different clays including (i) Montmorillonite clays with different principal cations and (ii) non-swelling kaolinite. With these geo-architected samples the effect of clay type was also investigated, thereby examining the effect of chemical composition. The final 2D image data of the different CB rock analogues after drying for systems containing 20% Montmorillonite (i) K10, (ii) STX-1b, (iii) SWy-3, and (iv) 5% Montmorillonite K10 (Supplementary Note 10 and Fig. 9b–e), the base case for the simulated data (Fig. 9a), and the CB-SKAO sample (Fig. S4) show that damage is only observed in structures whose constituent materials exhibit high shrinkage rates. For example from TGA-DSC (Supplementary Note 7) the Montmorillonite clays had a shrinkage rate of 1% mass loss per minute up to 100 °C for a heating rate of 20 °C/min).

No quantifiable damage was observed in the microstructure of the CB-SKAO sample (Fig. S4). Conversely, differences in the extent and distribution of damage were only observed in CB samples that contained some percentage (5% or 20%) of Montmorillonite, a swelling clay, which was the only material that experienced substantial mass loss during the TGA-DSC experiments. The effect of reducing the percentage of clay distributed in the matrix is evident by the drastic reduction in the $N_c$ and extent of damage, which is identified by a decrease in the percent volume of cracks from 8.93% (CB-SK10) to 0.24% (CB-SK10-5) in the microstructure of the CB synthetic rock that contained Montmorillonite K10 (Fig. 9e and Tables S4, S5, and S6). The extent and distribution of damage in the CB-SWY geo-architected rock (Figs. S5 and 9d), is localized within the body of the core, forming a network that intersects at the top center of the sample, which is different from the base case of the PD data (Fig. 9a), and the CB-SK10 sample (Fig. 9b,c) where cracks extend from the edge of the sample moving inward to the center or CB-STX (Fig. 9c).

The crack networks induced by differential shrinkage during moisture loss were analyzed using the Ridge Detection module plugin^21^ in FIJI^22^ that detects curvilinear structures and estimates features such as width, length, number of intersecting (jointed) cracks, and crack locations. The algorithm was applied to 2D binary images (both in the map and side views in Figs. S6 and S7)^21^ to quantify features of the crack networks. Marked differences in $N_c$, the number of cracks and the percentage of cracks per unit area ($\text{mm}^2$), $c_a$, correlate with the observed damage for both the map and side views. High values for both parameters were obtained, for example in the map view for the PD data ($N_c$ = 1022, $c_a$ = 98%), the CB-SK10 ($N_c$ = 197, $c_a$ = 18%) and the CB-STX ($N_c$ = 601, $c_a$ = 58%) samples which also experienced the most damage and the more complex fractured networks. CB-SWY ($N_c$ = 88, $c_a$ = 8%) and CB-SK10-5 ($N_c$ = 4, $c_a = < 1\%$) had lower $N_c$ and percentages of $c_a$. While the intersecting cracks of the PD binary data do not appear to be as fractal as the experimental data (Figs. S6, S7 and Tables S5, S6) the average values of the aperture (map = 0.562 and side = 0.263 ) and length appear (map = 1.446 and side = 0.477 ) were similar to experimental data (e.g. CB-SK10 map = 0.459 and side = 0.270 apertures and map = 2.712 and side = 1.244 lengths). The aperture is the distance between the 2 crack surfaces, and length is the distance along the selected ridge points of the crack identified by the algorithm. Further discussion and details regarding the methods used (Supplementary Note 9) and the crack network analysis are provided in Supplementary Note 10, and Fig. S8 where the distribution of the average crack lengths, apertures, and maximum aperture extracted from the 2D center image slice for each sample type for both the map and side views are shown.

### Damage distribution comparison

The comparison between the PD simulations and and the experimental data is a crucial step in the process of determining the degree to which the model accurately represents the experimental data. To quantify the similarities between the complexity of the PD binary damage network and the experimental crack networks a fractal analysis plugin in the FIJI open source image processing software, Fraclac^23^, was used. Fractal analysis is a method commonly adopted to perform quantitative texture and morphological analysis of image data. The fractal geometry characterizes the scaling structure of a surface by a number D^24^, referred to here as $D_F$, which is the fractal dimension. $D_F$ is an index of how detail changes with resolution based on the notion of some dimension, and also how a structure covers space (for this study between 1D and 2D). Prior to analyzing the data with Fraclac, all images were scale calibrated, thresholded to create binary images, and converted to 8-bit images using FIJI^22^. The simulated data were also converted to binary data and the low to no damage areas (cooler colors) were removed. The local connected fractal dimension (LCFD) analysis method was then applied to quantify the degree of complexity of the different crack networks. Every locally connected fractal dimension is associated with a single pixel, which can be used to produce a map of $D_F$ in the image space (Fig. S10). The LCFD method estimates the local mass scaling properties, which is the increase in the number of pixels forming the image of the damage network (i.e. a mass increase) within the confines of masks (boxes) increasing in size. Essentially, LCFD measures the total number of pixels ($N(\epsilon )$) that are locally connected within a box of increasing size ($\epsilon$) that is centered at some point^25^.

**Figure 10:**
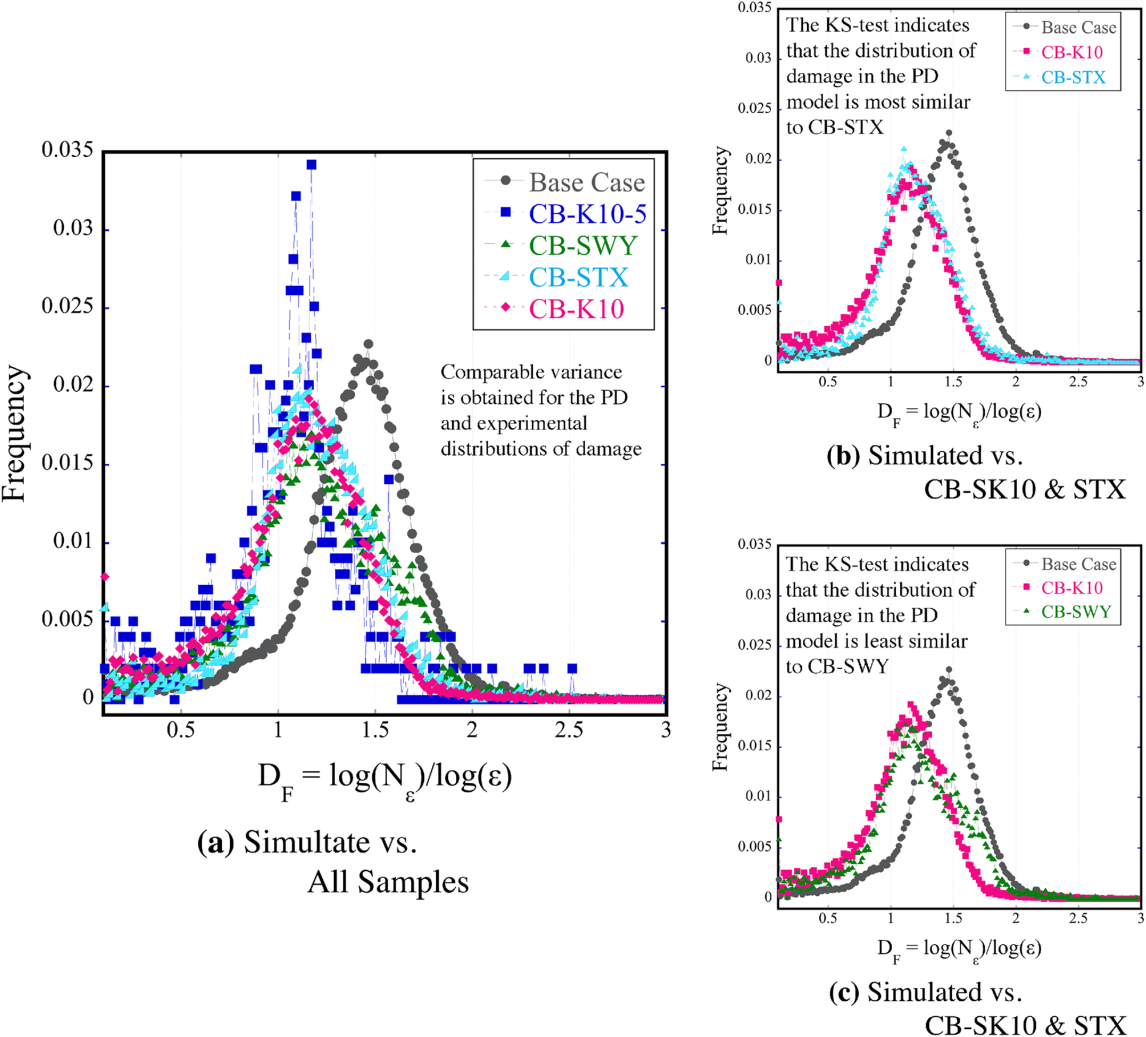
The mean distribution of the fractal dimension $D_F$, is a measure of the change in crack density with a change in scale estimated for each sample. Here a comparison (**a**) of the PD data versus the damage distribution in all experimental samples is plotted, along with (**b**) histograms for the PD data, and experimental samples CB-K10 and CB-STX, and (**c**) the distributions for Cb-SK10, Cb-SWY, and the PD data.

**Figure 11:**
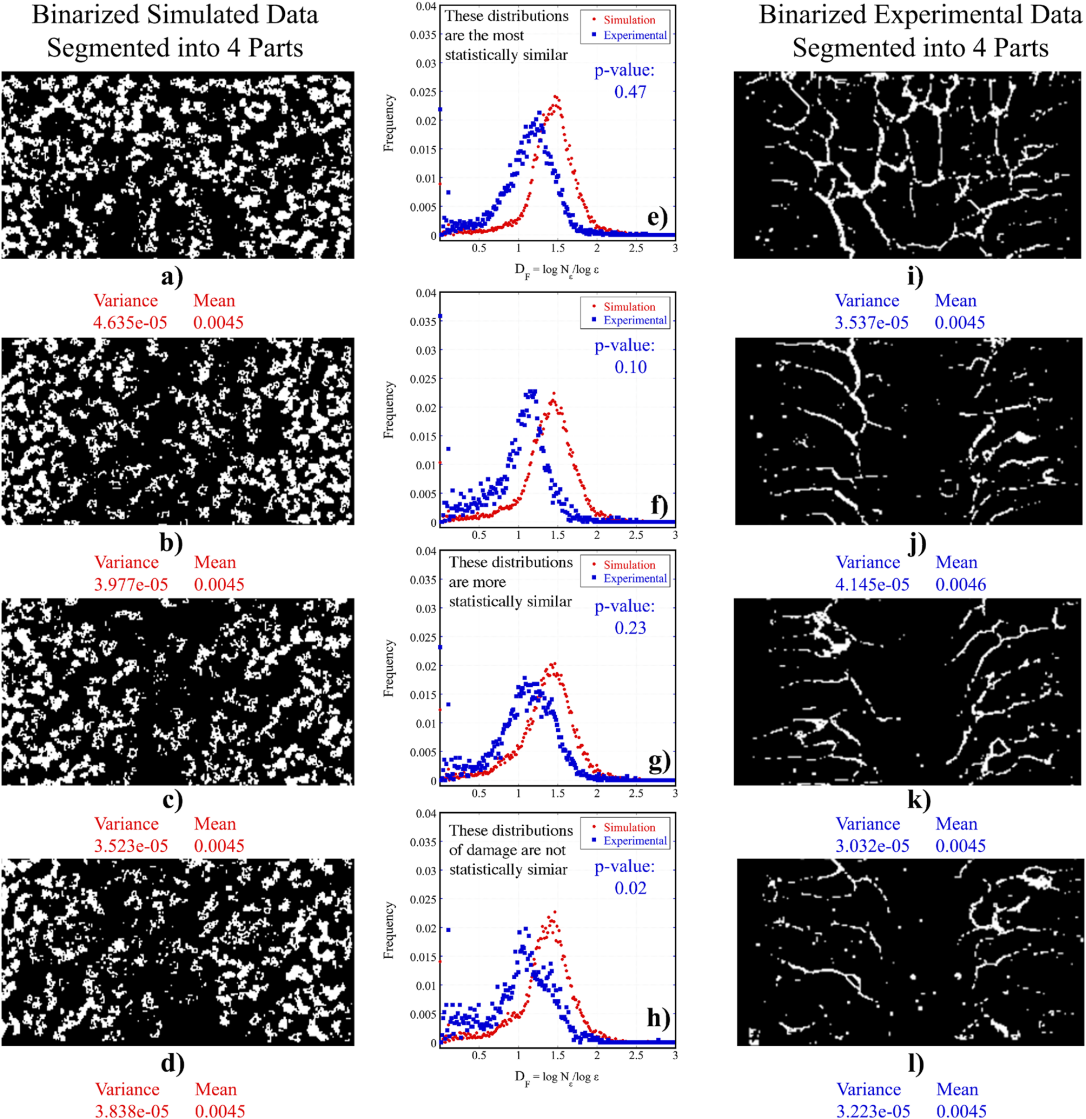
Sections of the simulated (**a**–**d**) and experimental data (**i**–**l**), plotted with histograms that compare the average distribution of the fractal dimension in each section for the simulated versus the experimental damage and estimated variances, means, K-statistics and p-values for each comparison.

The average distribution of ${D}_F$, which describes the degree of complexity over the image, is shown in Fig. 10 for all samples, and Fig. 11e–h for segments of the simulated versus the experimental data. The box counting method was used for analysis, where a box completely filled with cracks would result in $D_F = 2$. Though the peak location for the simulated data does not quite match with the peak location of the histograms for experimental data, the variance of the histograms are quite similar and the means of these distributions are as well albeit to a lesser extent (Table 1). The exception is the CB-SK10-5 sample. The PD data has a larger distribution that tends towards higher dimensional features, and is visibly less fractal which is conveyed by the skewness (1.52) of the mean distribution for the $D_F$ of the PD data. This indicates that more areas exist where boxes of increasing size $\epsilon$ can be approximately filled with “damage”. The observed experimental crack networks on the other hand, are more fractal due to coalescing cracks.

**Table 1:** The variance, and mean for all data, and the Kolmogorov–Smirnov statistics for the smoothed Base case distribution in comparison to the distribution of the other samples.

Data type	Variance	Mean	K-statistics	p-value
PD data	4.114e−05	0.0045		
CB-SK10	3.394e−05	0.0045	0.098	0.23
CB-STX	4.119e−05	0.0045	0.054	0.91
CB-SWY	3.094e−05	0.0045	0.116	0.10
CB-SK10-5	5.330e−05	0.0048	0.214	6.44e−05

To verify similarities between the mean distributions obtained for each sample in comparison to that of the PD data (Table 1), the two-sided Kolmogorov–Smirnov (KS) test in Python’s Scipy package was used^26^. This non-parametric test compared the underlying continuous distributions of two independent samples^26^. The obtained p-value provides a meter for which the null hypothesis is accepted or rejected. If the p-value is $< 0.05$, then the null hypothesis was rejected, which indicates that the two histograms were not from the same distribution. To implement the two-sided KS-test, the histograms are first approximated using a well-behaved interpolation method^27^, which generated curves that were a robust approximation of the original shape of the histogram. The null hypothesis was rejected when the simulated data was compared to the experimental CB-SK10-5 sample (Table 1). The null hypothesis was accepted for CB-SK10, STX, and SWY, where the comparison between the PD data and the CB-STX sample indicated that this distribution was the most statistically similar to the mean distribution of complexity of the PD Data. With a p-value = 0.23, similarities are assumed to exist between the histograms of the index of complexity for sections along the vertical axis of the PD 2D image center slice versus the vertical segments of the CB-SK10 experimental image data (Fig. 11).

The comparison between the PD data and CB-SK10 (Fig. 11) suggests statistical similarity in the mean distributions of complexity for the different segments. The null hypothesis for the first three segments were accepted with p-values of 0.47, 0.1, and 0.23 respectively, but the fourth segment (p-value = 0.2) was characterized by a distribution that was not statistically similar to the simulated data.

## Discussion

Drying shrinkage caused by the loss of adsorbed water is an important property of both cementitious and swelling clay materials. It can induce stresses that alter the structural and mechanical properties and long-term resilience of materials through the emergence of damage in the matrix from crack formation or debonding at the interfaces. The extent of shrinkage (or swelling) experienced by the embedded clay varies with the amount and type of clay minerals, the stress conditions, and the permeability^28,29^. The long-term structural integrity of clay-bearing porous media, which are often used as repositories for high level nuclear waste or energy products and for which the CB samples are proposed as an analogue, are particularly influenced by the state of moisture in the microstructure. The nucleation of fractures and propagation of the crack networks depends on the initial moisture condition (the amount of fluid in the porous medium), moisture loss behavior (the rate at which the sample dries), the permeability of the sample, and the curing time that controls the initial moisture condition and the strength as well as other mechanical factors.

As observed in the experimental data (Figs. 1a and 2a), no evidence of structural alterations is evident in the numerical experiments that contain only mortar (Figs. 2e and 6a). This validated both the approach and the material properties used for the matrix material. Additional analysis revealed that no structural changes occurred in the experimental CB sample containing a non-swelling Kaolinite (Fig. S4), which had a dehydration rate comparable to OPC.

Corroboration between the experimental data and numerical simulations is shown by structural transformations from intact bodies to damaged regions observed through 3D X-ray microscopy of the localized clay structures (one or multiple clay balls) (Figs. 1b,c, 2b,c) and the CB samples (Figs. 1d and 2d), which possessed qualitative similarities to the crack patterns and the damage distribution simulated with the PD continuum models (Figs. 2f–h, 6b–d). The PD approach was further validated through quantitative analysis of the simulated and experimental data which examined the features of the damage networks (aperture, lengths, $N_c$, etc.), the distribution of damage, and the similarities (and differences) between the distributions obtained through fractal analysis. The fractals are described by a recursive relationship with the complexity represented by the fractal dimension that determines how densely a fractal fills the space in which it occurs. As a result the fractal dimension expresses the tendency of the samples (simulated and experimental) to crack within the image space. Similarities between the histograms of mean distribution of complexity for the PD data and the experimental crack networks (Figs. 10 and 11) which were also characterized by higher percentages of cracks per unit area $c_a$ (Tables S5 and S6) and therefore a larger percent of damage, were uncovered using the KS-test (Table 1 and Fig. 11). Crack coalescence plays an important role in the propagation of damage, the fracture network and rock deformation^30^, and though jointed cracks were extracted for the PD data base case, the intersecting cracks were not similar to the experimental data. Furthermore, the observed spatial distribution (Fig. S10) and distribution of aperture (Fig. S7) were comparable despite the evident differences in cracks coalescence which may be related to the short crack lengths (Tables S5 and S6) extracted from the simulated data. The poor branching of these crack could be rectified by incorporating more nodes in the simulations to better replicate the experimental microstructure.

The process of fracturing is a critical phenomena in geophysics that results in an array of damage and crack morphologies precipitated by varying mechanisms and processes. The numerical experiments highlighted several parameters that have profound effects on the resulting fracture networks formed during drying. Comparisons of the simulated damage in the CB geometries are presented in Fig. 7. In particular, a comparison of Fig. 7b,c,g,h with the base case in Fig. 7a demonstrate that the clay and mortar shrinkage parameters have a dominant effect on the amount of fracturing relative to other evaluated parameters. The clay shrinkage values were focused on a base case of 20% shrinkage, and either doubled to 40% or decreased to 1%. Values as low as 0% were not evaluated, as is it clear that some amount of shrinkage is occurring in the clay. Similarly, the mortar shrinkage value was increased to 1% from a base case of 0.1%. Additionally, no mortar shrinkage was considered (0%) as a lower bound. Figure 8c illustrates the impact of clay shrinkage where a small shrinkage value of 1% results in an increase in the number of slightly damaged nodes (damage $< 0.05$), indicating the potential nucleation of microcracks. Additionally, high levels of clay shrinkage (40%) and mortar shrinkage (1%) increased the amount of damaged nodes in the range of 0.25–0.40, indicating the formation of larger and more interconnected cracks. Based on these variations, differential shrinkage appears to be the dominant driver for fracturing in composite materials such as the clay-mortar system.

The critical stretch parameter, which is related to the fracture energy and the strength of the material, had a mixed effect depending on which portion of the model was strengthened or weakened. In the parameter study, the critical stretches for clay, mortar, or the interfaces were varied. Specifically, twice and half the nominal values were selected as the upper and lower bounds for each material interaction. Ultimately, strengthening or weakening the matrix (mortar) material significantly impacted the amount of damage, (see Fig. 7d,i) with only a marginal effect on the amount of fracturing when the critical stretch is varied in the clay or across the interface (see Fig. 7e,k). Generally, the clay and the clay-mortar interfaces fracture earlier and faster than the mortar, so that changes to the low strength clay and clay-mortar interface have limited effect on the crack patterns in these systems. Decreasing the critical stretch in the mortar, analogous to weakening the cement, from $2\times 10^{-3}$ to $1\times 10^{-3}$ (or an absolute change of 0.001) encourages fracturing in the mortar region. Figure 8b identifies that only the change in the critical stretch of the mortar alters the damage distribution, with more damage in the 0.3–0.4 region.

These results and observations are consistent with the analysis of the interface pressure developed by spherical inclusions in a confining matrix. Considering the analytic elastic solution for the interface pressure, p, of a spherical inclusion in a matrix^31^

1 $$\begin{aligned} p = \frac{(\alpha _\text {matrix} - \alpha _\text {pore}) q}{(4 G_\text {matrix})^{-1} + (3 K_\text {pore})^{-1}} \end{aligned}$$

enables us to interpret the sensitivities of fracturing to the mechanical properties of the matrix and pores. If a limiting (negative) interface pressure is associated with interface fracture strength, it is clear that the difference in shrinkage parameters $(\alpha _\text {matrix} - \alpha _\text {pore})$ is the main determining factor; however, this effect is scaled by the local moisture concentration *q* and the effective elastic modulus $({(4 G_\text {matrix})^{-1} + (3 K_\text {pore})^{-1}})^{-1}$ comprised of the shear modulus of the matrix $G_\text {matrix}$ and the bulk modulus of the pore $K_\text {pore}$. Increasing the differential shrinkage or making the components of the mixture stiff should lead to fracturing from de-cohesion of the pore material from the matrix.

Another parameter that can alter the fracture of the clay-mortar composite is the interface moisture diffusivity (see Fig. 7n,o). The diffusivity was varied over the same magnitude as the critical stretch parameter, with twice and half the base case used as the upper and lower bounds, respectively. This variation included an absolute change in the diffusivity parameters of between $5 \times 10^{-4}$ to $5 \times 10^{-2}\; \text{m}/\text{s}$. Interestingly, varying the diffusivity in either the clay or mortar has a less pronounced effect (see Fig. 7l,m,p,q). Quantification of the amount of damage, i.e. broken bonds in the PD model in Fig. 8, shows that change does not occur in the damage distribution in the model but rather there is an increase in the number of nodes with low (0.1–0.2) damage, indicating that 10–20% of the bonds have been broken. Since the diffusivity in the clay is already quite high, the moisture diffuses quite quickly in the highly disconnected clay regions; however for the drying front to propagate further into the material it must pass through regions of mortar. These results are consistent with the experimental observations, as the moisture loss is quickest in the CB samples where the clay which often has a high moisture content prior to dehydration, causes the rock to initially retain more water. The CB-SK10 geo-architected rock, and other CB samples containing montmorillonite (CB-SWY and CB-STX) lose more water over the same period of time than the reference sample where the average rate per hour of moisture loss is greater than three times that of the reference.

The initial damage analysis for samples LICI, MSCS, and CB-K10, (Fig. 5), and initial strength data (Table S4) for the experimentally geo-architected samples along with the results from the additional CB sample investigations (Figs. 9, S4, and S5), quantitative analysis (Supplementary Note 10 Figs. S9, S10, and S11), PD simulations and experimental and simulated parameter analysis (Figs. S6, S7, and S8 and Tables S5 and S6), affirm that the presence of Montmorillonite clay (a highly shrinkable material) in the CB and the LICI samples significantly alters the cracking behavior, affects the drying paradigm, diminishes material properties such as strength, and increases porosity and conceivably permeability. In both the numerical and experimental scenarios swelling clay minerals are shown to be a crucial parameter for the observed damage, as varying degrees and distribution of damage arise when the swelling clay is distributed or localized within the microstructure with its relatively high moisture content and differential shrinkage rates. Prior to drying both the swelling-clay and the non-swelling clay work to keep the matrix intact. As drying occurs, the stresses induced by the differential shrinkage rate of the swelling clay which is higher than that of the background homogeneous medium leads to damage that does not occur with non-swelling clays. The materialization of cracks in the presence of distributed swelling-clay (a material with high initial moisture content and shrinkage rates) is a clear indication that the same phenomena occur in the experimental systems and the numerical models. This understanding is reinforced by the comparison of the additional CB samples which included a geo-architected rock that consisted of 20% distributed calcined Kaolinite, which has minimal differential shrinkage (Figs. 9, S4, and S5). This investigation demonstrated that the presence of constituent components with the propensity to shrink disproportionately within a homogeneous background matrix will result in damage to the background medium. This is especially apparent due to the extensive distribution of damage in the PD model that consisted of material with high differential shrinkage, and as cracks were only observed in the samples containing Montmorillonite, even in samples with decreased clay volumes (5% distributed Montmorillonite, Fig. S10e). Moreover, no structural alterations were observed in rock analogues containing calcined kaolinite (non-swelling clay) with a low initial moisture content, minimal dehydration, and negligible differential shrinkage rates (see Fig. S4).

Overall, differential shrinkage in swelling clay-mortar geo-architected samples was investigated through direct experimental evaluation and corresponding PD simulations. Three different geometries consisting of a single large isolated clay inclusion (LICI), multiple small clay spheroids (MSCS), and distributed clay (CB) minerals in a mortar matrix were evaluated, along with the base case reference sample that consist of mortar only which contained no clay material (M). The CB geometry was expanded to consider CB samples with different types of clay and a lower percentage of clay. The crack patterns in the PD models were qualitatively and quantitatively consistent with the experimental results for all the geo-architected sample types (LICI, MSCS, and CB), investigated, indicating that PD models can be used to efficiently investigate damage and fracturing in materials that exhibit dissimilar hydration properties. Through the use of a parameter study, the shrinkage properties of the clay and mortar, and the critical stretch (strength) of the mortar was found to have the largest impact on the amount of cracks, identified qualitatively through crack patterns in Fig. 7 and by increases in damaged nodes, Fig. 8. The increased damage indicates the formation of larger cracks within the structure. The strength at the clay-mortar interface and the water diffusivity did not significantly change the amount of fracturing observed over the ranges these parameters were varied. We can therefore surmise that the differential shrinkage rates strongly influenced the fracturing in these clay-mortar materials through decohesion and subsequent increase of stress concentration around the pores. Future work will focus on predictive simulations of fracturing in complex composites and polymineralic materials.

## Methods

### Experimental methods

#### Specimen materials and fabrication

Samples for moisture loss experiments of length $76.2\; \text{mm}$ and diameter $38.1\; \text{mm}$ were fabricated using (OPC)^9^, Ottawa sand^8^, and commercially available montmorillonite K10^10^, which were obtained from the sources listed in the Supplementary Note 1, Table S2. Four synthetic rock types were architected with controlled features, geochemistry and/or mineralogy that promote repeatable experimental behavior. The reference sample (and baseline mixture) is comprised of OPC and sand to create a mortar. The clay bearing synthetic rocks were structured as (i) a sample with one localized assemblage of clay particles, (ii) four bodies of clay randomly placed in the background mortar matrix, and (iii) clay particles distributed throughout the mortar framework constituting 20% of this sample type. Information regarding the materials used and fabrication protocols are given in the Supplementary Note 1 and Supplementary Note 2.

#### Drying experiments and damage characterization with in-situ 3D X-ray microscopy

Drying experiments were conducted for all sample types within a (Zeiss Xradia 510 Versa) X-ray Microscope in ambient conditions of humidity and at a temperature of 28 °C. To examine crack nucleation and damage propagation in the geo-architected synthetic rocks during moisture loss, a Zeiss Xradia 510 Versa X-Ray Microscope was used to perform 3D imaging and in situ 4D investigations. All samples were imaged immediately out of the curing environment (which signifies the start of the monitoring period) at a voxel edge length resolution of  $40\; {\upmu} \text{m}$, and re-imaged intermittently over the course of 6 days. The exact settings used to acquire the X-ray microscopy data for each sample type are given in the Supplementary Note 8.

#### Unconfined compressive strength (UCS) testing

Under displacement (strain) controlled conditions at a constant loading rate (see Supplementary Note 4), the unconfined compressive strengths (UCS) of undrained samples were obtained using an Instron loading machine for representative specimens of each sample type at the onset of the monitoring period.

#### Water loss monitoring

In unconfined ambient conditions (ambient temperature and humidity), the moisture lost (weight lost) in one representative distributed clay and one reference geo-architected rock, were automatically recorded per minute by an Ohaus SPX2202 scale for a period greater than six (6) full days. Additional information is provided in Supplementary Note 5.

#### Thermal analysis of fabrication materials

Thermogravimetric analysis with differential scanning calorimetry (TGA-DSC) is a robust reliable experimental test for the characterization of the thermal behavior of materials, and is used to analyze the dehydration rates of the different clay types. The OPC, commercially obtained Montmorillonite K10, Montmorillonites SWy-3 and STx-1b, and a calcined Kaolinite (metakaolin obtained from IMERYS Group) were examined using a TGA-DSC gas delivery SDT Q600 system from TA Instruments in a nitrogen (N_2_) gas atmosphere with a heating rate of 20 °C/min from ambient temperature up to 1000 °C, which is the final temperature. Approximately 40 mg of each material was tested under such a wide temperature range to distinguish and evaluate water loss from the microstructures. Additional notes on this method are provided in Supplementary Note 6.

#### Damage quantification and comparison

The acquired data were initially examined using Dragonfly Pro software, Version 2020.2 for [Windows] from Object Research Systems (ORS) Inc.^13^. Initial data analysis involved thresholding segmentation to generate regions of interest (ROIs) for the extraction of different networks (unconnected round pores and connected cracks), quantification of sample properties (length, diameter, volume, porosity, damage), and comparison of the different sample types. FIJI is then used to perform the quantitative analysis specifically examining the experimental microstructure and the PD data. This is done to quantitatively validate the approach of using PD to model crack formation and the evolution of damage in the microstructure during dehydration, driven by the differential shrinkage rates between highly shrinkable material and a fairly homogeneous background matrix. FIJI^22^, an open source image analysis and processing package, is used along with specific plugins such as Fraclac^23,25^ and Ridge Detection^21^. FIJI is used to perform thresholding segmentation and binarize the image data. Then, the Ridge Detection plugin is utilized to detect, extract and characterize the features of damage, i.e. the crack network such as the number of single and/intersecting cracks ($N_c$), aperture, and percent crack per unit area $\text{mm}^2$ ($c_a$) which is defined by the number cracks divided by the total area (i.e. $c_a = N_c$/$\text{mm}^2$) of the sample. This plugin is based on a detection algorithm formulated by Steger^21^ that is used to detect ridges and lines, here it is used to detect and characterize the spatial dimensions of structural inhomgeneities. The Ridge Detection plugin was also be used to create binary image date from the crack overlay generated for the PD or experimental data.

For a quantitative description of damage for both the experimental and PD data, the Fraclac plugin which performs morphological fractal analysis is used to investigate the complexity of the data using the box counting method which is applied by overlaying a grid of boxes on the crack image and counting the number of boxes within the confines of the image domain, then repeating this process for boxes of different sizes. Fraclac performs fractal analysis on each binarized 2D center slice image for all samples and is applied to four segments of the PD and experimental CB-SK10 image data. In the FracLac plugin, the linear connected fractal dimension (LCFD) analysis was applied with a scaling method that used odd sized boxes for box counting. The grid of odd sized boxes used to sample 2D binarized images (Fig. S9) and results in a color-coded LCFD scan that highlights the different modes of local variation (Fig. S10) where cooler colors relate to areas of lower values of $D_{LC}$, the locally connected fractal dimensions which is estimated with sub-pixel accuracy. The LCFD method performs a linear regression of the logarithm of the mass of pixels N($\epsilon$) in a box of size $\epsilon$, divided by the logarithm of $\epsilon$ and is used to find the scaling relation^25^. The mean distribution of complexity ${D}_F$ which is an index of how detail changes with resolution based on the notion of dimension is also obtained for all samples and plotted in Fig. 10, where higher values of $D_F$ can be interpreted as increasing dimensional complexity as the fractal dimension increases. More information regarding the use of the plugin can be found in Supplementary Note 9.

To verify the similarities between the mean distributions of complexity obtained from the LCFD scanning module in Fraclac for each experimental sample and the distributions estimated for the PD data (Fig. 11, the two-sided Kolmogorov–Smirnov (KS) test in Python’s Scipy package is used^26^. This non-parametric test compares the underlying continuous distributions *F*(*x*) and *G*(*x*) of two independent samples^26^ and provides K-statistics and p-values. The p-value obtained is a meter for which the null hypothesis that the sample datasets are identical and therefore come from the same distribution (i.e. $F(x) = G(x) for all x$) can either be accepted or rejected. Assuming a threshold, i.e. the probability of being rejected, is equal to 0.05 (which is a practical value for most applications), if the p-value is < threshold then the null hypothesis can be rejected and the two histograms are not from the same distribution. If the KS statistics is small or the p-value is high, then the null hypothesis cannot be rejected^26^. Prior to implementing the the two-sided KS-test, the histograms are first approximated using a well-behaved interpolation method^27^ that generates a curve to exactly match the given slopes of the points of the input data and therefore retains a robust approximation of the original shape of the histogram. The KS-test is then applied to the interpolated data comparing the distribution for the simulated base case to all the CB experimental samples, and additionally comparing the CB-SK10 sample to the entire CB sample group. From the KS test, and the subsequent acceptance or rejection of the null hypothesis based on the p-value, similarities between the PD and experimental data can be determined. Additional information regarding image and damage analysis techniques, software, and the data characterization processes are elaborated on in Supplementary Note 9.

### Computational methods

A model of differential shrinkage and resulting damage occurring during the dehydration of porous media was developed to correspond to the experimental conditions. The drying is simulated with a diffusion model which is similar to the drying analysis explored by Sherwood^32^. While this is certainly oversimplified, it provides a reasonable description of drying without complicating the model. To incorporate damage resulting from fracturing and crack coalescence into the model, the non-local theory of PD^4^ is employed. A key feature of the PD model is the lack of spatial derivatives in the governing equations. This feature avoids regularity issues arising from discontinuities such as cracks. There are two main formulations of PD: bond-based PD^4^ and the more general state-based PD^33^. This work focuses on bond-based PD wherein each material point interacts directly with material points within a region about the material point called the neighborhood. In this work, the neighborhood of a material point is taken as the region within a fixed distance $\delta$, referred to as the horizon, of the material point. In the reference configuration, a material point is assumed to have a connection called a *bond* to every material point within its neighborhood. Damage is introduced into the model by allowing these bonds to break irreversibly according to a criterion, which in this work is a stretch threshold for the bond. When bonds break their loads are transferred to surrounding material points and can result in the progressive breakage of bonds. In order to conduct numerical experiments, the model was implemented into the massively parallel open-source code Peridigm^34^.

#### Drying model

Given the range of pore size and geometries present in cement, mortar, and clay, we used phenomenological modeling to describe the evolution of the drying front in these systems. In the literature it is common to focus extensively on the physics behind the drying process, including features such as hydroionic transport^35^, capillary pressure^36,37^, and structure of the porosity^38^. In contrast a simplified approach is applied here that describes a diffusion driven drying front that propagates into the material and effects change in the mechanical properties of the material. Numerical details are included in Supplementary Note 11.

#### Mechanics model

The mechanics portion of the simulation employs an adaptation of the prototype microelastic brittle (PMB) peridynamic model^5^. For convenience in describing the model, we utilize a standard shorthand:

2 $$\begin{aligned} \varvec{\xi }:= \mathbf {x}' - \mathbf {x} \quad \text {and} \quad \varvec{\eta }:= \mathbf {u}(\mathbf {x}',t)-\mathbf {u}(\mathbf {x},t), \end{aligned}$$

where $\mathbf {x}'$ and $\mathbf {x}$ are material points, $\mathbf {u}$ is the displacement field, and *t* is time. The equation of motion is then given by

3 $$\begin{aligned} \rho (\mathbf {x}) \ddot{\mathbf {u}}(\mathbf {x},t) = \int _{B_\delta (\mathbf {x})} c s(t,\varvec{\eta },\varvec{\xi }) \mu (t, \varvec{\xi }) \frac{\varvec{\eta } + \varvec{\xi }}{| \varvec{\eta } + \varvec{\xi }|} d \mathbf {x}', \end{aligned}$$

where $B_\delta (\mathbf {x})$ is a ball of radius $\delta$ about $\mathbf {x}'$, *c* is the bond stiffness, *s* is the bond stretch, and $\mu$ is a binary-valued function that is 1 for an intact bond and 0 otherwise. The criterion in this work for a bond to be intact is the stretch *s* never exceeding a critical stretch $s_c$. The PD parameters are calibrated with physical properties. Specifically, the bond stiffness is related to the bulk modulus through the relation

4 $$\begin{aligned} c = \frac{18 K}{\pi \delta ^4}, \end{aligned}$$

and the critical stretch is related to the fracture toughness through the relation

5 $$\begin{aligned} {s_c = \sqrt{\frac{5 K_{IC}^2}{8 K E \delta }}}. \end{aligned}$$

In order to incorporate drying into the mechanic model, the material properties *c* and $s_c$ are permitted to change with the moisture content $q(\mathbf {x},t) \in [0,1]$. Additionally, shrinkage is introduced into the model by defining the stretch as

6 $$\begin{aligned} s(t,\varvec{\eta },\varvec{\xi }) := \frac{|\varvec{\eta }+\varvec{\xi }|-\theta (\alpha ,q(\mathbf {x},t))|\varvec{\xi }| }{ | \varvec{\xi }| }, \end{aligned}$$

where

7 $$\begin{aligned} \theta (\alpha ,q(\mathbf {x},t)) = 1-(1-q(\mathbf {x},t))\alpha \end{aligned}$$

determines the equilibrium bond stretch, i.e., a bond will be in equilibrium if its length is a factor of $\theta (\alpha ,q(\mathbf {x},t))$ of its reference length. The parameter $\alpha \in [0,1]$ determines the degree of shrinkage. Further details of the mechanical model are described in Supplementary Note 12.

#### Numerical model geometries

The geometric structures utilized in the numerical experiments were constructed based on the experimental geo-architected rock samples which can be observed in Fig. 1a–d. The numerical experiments were all cylinders with radius $19.05\; \text{mm}$ and height $76.2\; \text{mm}$. The base material, mortar, is a homogeneous background matrix and clay is incorporated as an inclusion in three different geometries. The first two geometries where clay particles are incorporated are mortar with a LICI or MSCS. To produce realistic clay inclusions, the particles in the discretization were assigned to either mortar and/or clay through use of the 3D reconstructed tomographic image data obtained with 3D X-ray microscopy during the drying experiments. The full process of how this is accomplished is explained in Supplementary Note 3, and the resulting numerical geometries are shown in Figs. S1 and 6. The last geometry we consider is an analogue of the CB sample, shown in Fig. 1d. Since the experimental data could not resolve the individual clay particles in the background mortar, in the numerical counterpart clay spheres in a quantity representative of 20% were randomly distributed in the model geometry. The results of this are shown in Fig. S1c and also in Fig. 6.

#### Boundary conditions

Imposing boundary conditions in nonlocal problems is a notoriously difficult problem. In this study, the mechanics portion of the model allows the dynamics to be driven by the drying of mortar and clay, and the boundaries are free. In the diffusion/drying model, the boundary conditions are more complicated. We impose a Robin boundary condition analogous to Newton’s law of cooling:

8 $$\begin{aligned} -D \nabla c = h(c - c_\infty ), \end{aligned}$$

where $c_\infty$ is the ambient moisture and *h* is a parameter calibrated to the initial drying rate $-D_c \nabla c$. One method of imposing such conditions in the classical framework is to create a fictitious boundary on which a diffusivity $D_c^*$ is imposed. This approach is employed in this work wherein we create two fictitious regions outside the cylinder. The first is a layer where we impose the model (2) in Supplementary Note 11 with a diffusivity $D_P^*$. The second fictitious region is a layer of $\delta$ thickness outside the first fictitious layer on which we impose the boundary condition $q(\mathbf {x},t) = 0$. In the experiments the cylinders were capped on one end which prevented moisture loss through that surface. This is reproduced in the numerical experiments by setting the moisture diffusivity to zero for bonds that extend through that surface. The drying parameters in the numerical experiments include the solid mortar-mortar interface (1.0 $\times 10^{-3}\; \text{mm}^2/\text{s}$), mortar-clay interface (2.0 $\times 10^{-2}\; \text{mm}^2/\text{s}$), and clay-clay interface (5.0 $\times 10^{-2}\; \text{mm}^2/\text{s}$). The presence of the Robin boundary conditions also adds a second series of drying parameters between mortar-air (5.0 $\times 10^{-2}\; \text{mm}^2/\text{s}$), air-clay (5.0 $\times 10^{-2}\; \text{mm}^2/\text{s}$), and air-air (5.0 $\times 10^{-2}\; \text{mm}^2/\text{s}$), with air being the zone around the cylinder.

#### Numerical experiments parameters

The numerical experiments have a variety of parameters. The four geometries seen in Fig. 6 were discretized with nodes distributed over the domain with a spacing $dx = 0.25\; \text{mm}$ and perturbed up to $0.2\; dx$ in each of the cardinal directions. The perturbations were added to avoid issues related to crack dependence on the discretization. The horizon determines the range of interactions between material particles in all experiments and was set at $\delta = 0.75\; \text{mm}$. The time step in the moisture content simulation is $\Delta t = 5184\; \text{s}$ and 100 time steps were taken which corresponds to the six full days for the experimental simulation. The time step in the mechanics simulation is $\Delta t = 5.184\; \text{s}$ which corresponds to 1000 time steps between each loading from the moisture simulation. In addition, during the first 500 steps after a loading step, bonds were not permitted to break. This was done to prevent damage resulting from the initial transients in the application of the loading steps. Selection of specific modeling parameters for the clay and mortar properties is discussed in the Supplementary Note 14.

## Supplementary Information

Supplementary Information.

## Data Availability

Data is available upon request from the corresponding authors pending internal review and approval.
