# Supplementary material for: The effect of differential mineral shrinkage on crack formation and network geometry
Source: Sci Rep. 2022 Dec 23;12:22264. doi: 10.1038/s41598-022-23789-3 (PMC9789088; doi:10.1038/s41598-022-23789-3)
Supplement: Supplementary file 1 — Supplementary Information. [file 41598_2022_23789_MOESM1_ESM.pdf]

Supplementary Information: The Effect of Differential Mineral Shrinkage on Crack Formation and Network Geometry

Jeremy E. Trageser[1,*], **Chven A. Mitchell**[2,3], **Reese E. Jones**[4], **Edward N. Matteo**[3], **Jessica M. Rimsza**[5], **and Laura J. Pyrak-Nolte**[2,6,7]

[1]Center for Computing Research, Sandia National Laboratories, Albuquerque, NM, USA
[2]Department of Earth, Atmospheric, and Planetary Sciences, Purdue University, West Lafayette, IN, USA
[3]Nuclear Waste Disposal Research and Analysis, Sandia National Laboratories, Albuquerque, NM, USA
[4]Mechanics of Materials, Sandia National Laboratories, Livermore, CA, USA
[5]Geochemistry Department, Sandia National Laboratories, Albuquerque, NM, USA
[6]Department of Physics and Astronomy, Purdue University, West Lafayette, IN, USA
[7]Lyles School of Civil Engineering, Purdue University, West Lafayette, IN, USA

Supplementary Note 1

Materials and Sample Fabrication Protocols

Specimen Materials: The different sample types used in the experimental research were fabricated from ordinary Portland cement (OPC), Ottawa sand, and for the initial clay bearing sample, commercially available montmorillonite K10 which were obtained from the sources listed below:

Ottawa Sand: Material ID - Sand

This material does not swell and was procured from U.S. Silica (product name: SIL-CO-SIL). It is reported by U.S. Silica as a high-purity ground silica (99.5% SiO_2) with a particle size range from 45-250 μm, and a near-neutral pH[1].

Ordinary Portland Cement - Type II: Material ID - OPC

The ordinary Portland cement was obtained from LaFargeHolcim at the Alpena plant in Michigan. This material does not swell and is highly reactive in water. The Portland cement was used to construct the background matrix to provide a homogeneous carbonate rich structure in which to embed clay particles. A hydrated mixture of ordinary Portland cement and sand is referred to as mortar, and represents the reference sample. The experimentally obtained and reported pH range in water is 12 - 13. The chemical composition as reported by[2] is presented in Table S1, while the experimentally obtained elemental composition (%) is shown in Table S3.

Limestone	Gypsum	Calcium Oxide	Magnesium Oxide	Quartz
$CaCo_3$	$Ca(SO_4) \cdot 2H_2O$	CaO	MgO	SiO
0 - 15%	2 - 10%	0 - 5%	0 - 4%	0 - 0.2%

Table S1. List of the materials used to fabricate synthetic rock samples, the entity from which they were obtained, and a short material description

Montmorillonite (K10, SWy-3, & STx-1b

The Montmorillonite K10 material was purchased from Sigma Aldrich (product number: 69866), and is a commercially available montmorillonite that is manufactured as a K-catalyst. The clay material properties as reported by Sigma Aldrich[3] are, a surface area range of 220-270 m^2/g, and a pH range is 3–4[3]. For montmorillonite K10 the values for elements in the sample are obtained with XRF and are reported in Table S3. The elemental composition for a predominantly

Ca-montmorillonite (STx-1b) and a Na-montmorillonite (SWy-3) both obtained from the Clay Mineral Society at Purdue University in West Lafayette, Indiana are presented in Table S3. These clays which are minimally processed (milling of the material and drying at low temperatures) have comparatively different chemical compositions from the commercially available Montmorillonite K10. More information regarding the CMS clays can be obtained from references[4,5].

Calcined Kaolinite

Metakaolin, a calcined kaolinite obtained from IMERYS (product: Metakaolin MetaStar 501 which has a high level of pozzolanic activity) was also used to fabricate synthetic rock samples. This material serves as a representative non-swelling constituent material. The elemental compositions for this, as well as the other materials are shown in Table S3

Supplementary Note 2

Sample Fabrication:

With the materials presented above, synthetic rocks were architected with controlled features, geochemistry and/or mineralogy that promote repeatable experimental behavior[6]. All specimens were molded using cylindrical silicone molds of radius $\sim$ 19.05 mm and length $\sim$ 76.2 mm. The reference (mortar only) sample consisted of a 1:2.5 ratio by weight of cement to sand, and a water/cement ratio of 0.87. This high water cement ratio is purposely used to take advantage of the weakening effect of water and generate a reference synthetic rock with reduced intermediate strength and comparable water contents. The clay material was pre-soaked in water (for minimum of 24 hours) prior to adding it to the mortar mixture (cement and sand). The pre-soaking step is performed to minimize loss of available water needed for cement hydration to the clay particles. Incorporating un-calcined, montmorillonite clay particles in OPC mortar creates a situation where there is a competition for water between the reactive constituent materials. Montmorillonite can adsorb water into its structure and swell or shrink depending on the temperature, availability of water, and the chemistry of the fluid in the in situ environment[7,8]. Ordinary Portland cement however needs a sufficient amount of water to hydrate and harden[7,9]. To fabricate the reference cylindrical samples, first the dry ingredients are blended together, then deionized water is added to the dry ingredients and mixed until a paste is formed. This paste is then deposited into the cylindrical mold and vibrated for compaction and to release trapped air. The clay bearing synthetic rocks are structured as i) a sample with one clay sphere where the clay ball occupies approximately 11% of the volume , ii) the four clay bodies randomly embedded in the reference mortar matrix where the multiple clay bodies are expected to occupy approximately 5-6% of the sample volume, and iii) 20% clay distributed throughout a mortar framework. The small (multiple spherical clay structures - MSCS) and large (single clay assemblage - LICI) clay inclusions were shaped by freezing the pre-soaked clay material (for a minimum of 24 hours) using silicone ice molds with approximate diameters of 6.5 *mm* and 13 *mm* respectively. To fabricate the clay-bearing samples i) and ii), first the reference mortar mixture is created as mentioned above. A portion of this mixture is then deposited into the cylindrical mold at a depth, dependent on where the body of clay will be positioned (e.g. for a single large clay ball this would be up to the halfway point of the mold). The first layer is always vibrated for 15 seconds prior to the addition of the frozen clay ball. After the frozen clay is positioned, additional layers are added as needed until the mold is filled. Then the mold is vibrated one last time for 15-20 seconds. Long periods of compaction with vibration should be avoided when working with frozen clay material. For the iii) distributed clay sample (CB), the slurry containing 20% of clay powder by weight (in relation to the total weight of cement and sand) is added to the reference mixture and blended until evenly distributed within the paste. The paste is them added to cylindrical molds and compacted with vibration. All blending was performed with a KitchenAid stand mixer and the compaction and removal of air during the molding of the samples was performed with a small square dental lab vibrator model oscillator. After the process of molding, the samples are placed in a humid chamber for 24 hours to harden and set. The samples are then de-molded, placed into zip-locked bags filled with deionized water which are then submerged in a hot water bath consisting of deionized water kept at 30°C $\pm$ 1°C for seven days. The different sample types are placed in sample specific curing tanks. When curing is complete the samples are removed and kept moist until the start of the experiment. The steps outlined for the CB (distributed clay bearing) sample were adopted to fabricate the additional CB samples which contained Montmorillonite (CB-STX, SWY and, CB-SK10-5 that is comprised of 5% clay) or calcined Kaolinite (CB-KAO). All CB samples except CB-SK10-5 contain 20% clay. The list of materials and where they were obtained is also summarized in Table S3.

Elemental composition with X-ray Fluorescence (XRF):

To perform elemental analysis of the materials used for fabrication, a Malvern Panalytical Epsilon 4 benchtop - Energy Dispersive X-ray Fluorescence (EDXRF) spectrometer equipped with an Ag anode X-ray tube was used with the Panalytical Omnian standardless data collection method. The measured fluorescent X-rays determine the abundances of elements, while the intensity of these rays is proportional to the concentration of the elements present in the sample[10]. No preparation methods were applied to the OPC or montmorillonite powder prior to analysis with the ED-XRF. While experimental data were obtained

for all materials, information from CMS[4,5], are provided for the SWy-3 and STx-1b Montmorillonites (Table S3).

	Sample ID	Company	Description
Ottawa Sand (Sil-Co-Sil)	Sand	U.S. Silica	Non-swelling material
Type II - OPC	OPC	LaFarge	Non-swelling material
Montmorillonite K10	K10	Sigma Aldrich	Swelling material
Montmorillonite STx-1b	STX	Clay Mineral Society	Swelling material
Montmorillonite SWy-3	SWY	Clay Mineral Society	Swelling material
Kaolinite (calcined)	KAO	IMERYS	non-swelling material

Table S2. List of the materials used to fabricate synthetic rock samples, the entity from which they were obtained, and a short material description

	SiO_2	Al_2O_3	Fe_2O_3	MgO	CaO	SO_3	Na
Type II – OPC (LaFarge)	19.7	4.5	3.0	2.4	63.6	2.6	0
Montmorillonite (K10)*	76	10.9	6.6	0.7	0.8	0	0
Montmorillonite (STX)†	70.1	16	0.7	3.69	1.59	0	0.2
Montmorillonite (SWY)†	62.9	19.6	3.35	3.05	1.68	0.05	1.53

∗ Experimentally obtained values
† Values reported by the Clay Mineral Society[4,5]

Table S3. Experimentally obtained and reported values for the elemental percent (%) composition of each material used for fabrication

Supplementary Note 3

Experimental and Simulated Sample Geometries

The model geometries (Figure S1) for the numerical experiments are obtained directly from the laboratory experimental sample geometries extracted from the 3D X-ray microscopy data. The reconstructed 3D tomographic image dataset was obtained at a higher resolution (at approximately $40\mu m$) than the numerical model (at approximately $250\mu m$). To produce realistic clay inclusions, the data are first segmented based on the existing constituents (mortar and/or clay) using Dragonfly Pro software, Version 2020.2 for [Windows] from Object Research Systems (ORS) Inc.[11], binarized, and downsampled to match the numerical resolution. The particles in the discretization were then assigned to the existing material types and locations directly from the experimental data by labeling each particle based on a nearest neighbor search over points in the experimental 3D image data. The resulting geometries are shown can be observed in Figure S1. The particles of clay dispersed within the matrix of the distributed clay sample cannot be visualized at the resolution of the experimental data. To efficiently replicate this geometry, we consider an analogue of the distributed clay, see example in Figure S1d. Instead, the numerical experiment randomly distributes clay spheres of radius $0.3mm$ so that approximately 20% of the particles in the discretization are clay. The resulting geometry is shown in Figures S1c. Figure S1a replicates the composition and geometry of the reference specimen, which is the mortar only sample, while Figure S1d reflects a sufficient analog of the distributed clay sample (because these particles cannot be resolved at the resolution of the 3D X-ray microscopy data), where the clay particles are randomly dispersed within a mortar matrix. Figures S1b and c reproduce the composition, and structure of the specimen with localized clay inclusions. The size, shape and location of the bodies of clay, with the exception of the distributed clay sample, are comparable to the laboratory data.

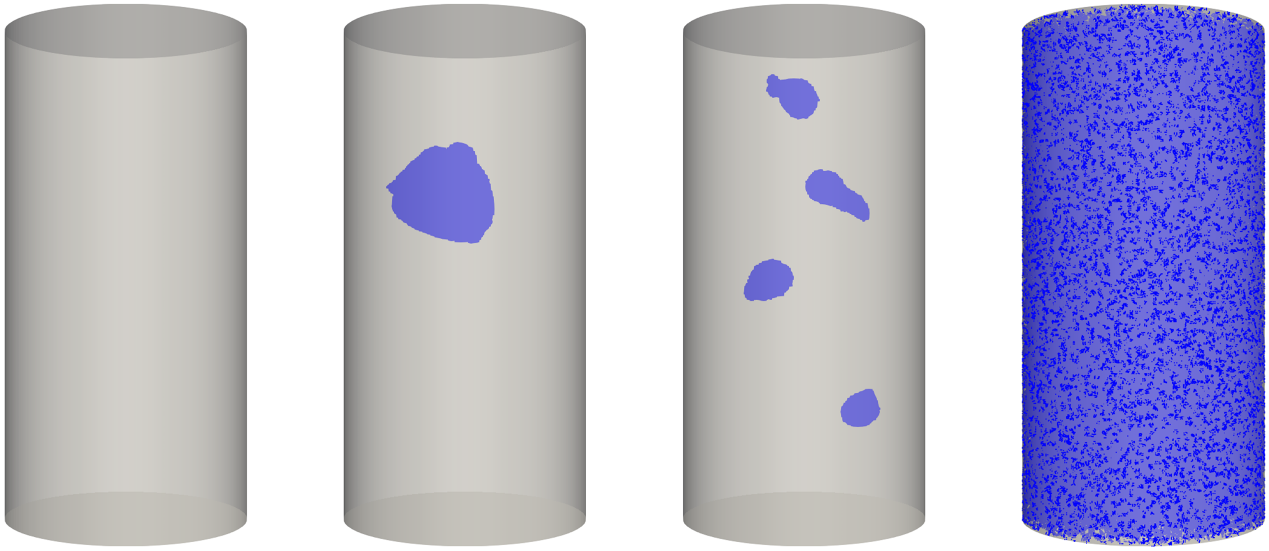

Figure S1. Model geometries for numerical experiments, where a) represents the reference sample, a homogeneous matrix, b) the sample with a single clay body, c) a model with multiple clay inclusions embedded in the homogeneous matrix, and d) clay particles randomly distributed within the homogeneous matrix

Supplementary Note 4

Unconfined Compressive Strength (UCS) Testing

Under displacement controlled conditions at a constant loading rate, the unconfined compressive strengths (UCS) of undrained samples were obtained for companion specimens of each sample type at the onset of the monitoring period. Six measurements were obtained of the length and diameter of the samples with a vernier caliper prior to deformation. To perform each test, the samples were mounted between two steel platens placed at the center of an Instron loading system, frame model model 59-R8100BTE, which was controlled with Bluehill 3 software. To minimize friction, sheets of Teflon were secured with Vaseline to the surface of each platen and the samples were placed between them. A minimum of three specimens for each sample type were tested. For all UCS tests the displacement rate applied was 0.08 mm per minute. The UCS was determined by dividing the maximum force achieved prior to failure by the cross-sectional area of the specimen. The average values are estimated using the UCS obtained from the deformation of a minimum of three samples of each geo-architected rock type. The average weight per geo-architected sample type is 160 grams for the localized clay sample structures (LICI & MSCS), 167 grams for the reference, and 148 grams for the samples with distributed clay (CB). Resulting UCS values are given in Table S4.

Sample Type	Total Porosity (%)	Total Crack Volume (%)	Average UCS (mpa)	Description of material
Mortar (M)	2.6	0	11.4	Non-swelling material
Multiple Clay Inclusions (MSCS)	3.6	0.7	11.05	Localized swelling material
One Clay Inclusion (LICI)	2.5	0.7	6.1	Localized swelling material
Distributed Clay (CB-SK10)	12	8.93	4.6	Distributed swelling material
Distributed Clay (CB-STX)	10.5	9.6	6.36	Distributed swelling material
Distributed Clay (CB-SWY)	6.8	4.58	1.15	Distributed swelling material
Distributed Clay (CB-SK10-5)	2.9	0.24	8.05	Distributed swelling material
Distributed Clay (CB-SKAO)	2.54	0	7.7	Distributed non swelling material

Table S4. Total segmented porosity which includes non-connected pores and connected cracks/voids, crack volume (cracks only), UCS, and a material description for each synthetic rock type after six (6) full days of dehydration

Supplementary Note 5

Moisture Loss Monitoring

In unconfined ambient conditions (ambient temperature and relative humidity), representative saturated geo-architected samples (newly removed from their respective curing tanks) were left to dehydrate on an Ohaus SPX2202 portable scale with a readability of up to 0.01 grams, that automatically recorded the weight of the sample per minute for a period greater than six (6) full days.

Supplementary Note 6

Thermogravimetric Analysis and Differential Scanning Calorimetry (TGA-DSC)

Thermal analysis was performed on OPC[2], and different Montmorillonites (K10[3], SWy-3[4], and STx-1b[4]), as well as the calcined Kaolinite. This method of analysis was undertaken to extract information regarding the water content, mass loss and the thermal behavior of these fabrication materials. Large differences in mass loss are indications of water content and may be used to understand the differences in material shrinkage. Approximately 40 mg of each clay and cement material was analyzed in an untreated, unprocessed state using an alumina TGA crucible where the weight is calibrated with an empty alumina crucible. Thermal analysis was performed using a TGA-DSC gas delivery SDT Q600 system from TA Instruments in a nitrogen (N_2) gas atmosphere with a heating rate of 20°C/min from ambient temperature up to 1000°C, which is the final temperature. A large temperature range is used to distinguish and evaluate the water loss of the minerals and OPC which can contain water in different forms. Several peaks relating to water loss are often observed in the TGA data measured for Montmorillonite clays; an initial endothermic peak which represents the loss of water held between the basal planes of the lattice structure (i.e. the swelling water) should occur between 100-250°C, a second endothermic peak between 600–700°C that corresponds to the loss of lattice water and dehydroxylation of the clay lattice $^{-}$OH group, and a third endothermic reaction at about 900°C which corresponds to the final breakdown of the clay mineral that is followed by an exothermic effect which is sometimes accompanied by the formation of spinel[12]. The third endothermic peak does not manifest in 1:1 dioctehedral kaolinites[12]. Features of pore water loss in montmorillonites do not appear in the thermal curves above 100°C since they lose most of the lattice water first[13]. In cements, any mass loss up to approximately 600°C is generally attributed to the loss of water, above 600°C mass loss is mainly due to the release of the CO_2[14].

Supplementary Note 7

Dehydration of Fabrication Materials and Clays

The thermal analysis of fabrication materials were performed and the data are presented in Figures S2a-e, with a comparison of the different mass loss curves explicitly shown in Figure S3. It is clear that the OPC and calcined Kaolinite (Figures S2d & e behave differently in comparison to the Montmorillonites. At the first peak the OPC (130°C) and Kaolinite (66 °C) lose 0.74% and 0.35% of their respective weights. The Montmorillonites, K10 which is used to fabricate the CB-SK10 and CB-SK10-5 samples, and STx-1b and SWy-3, all experience a first peak at temperatures greater than 100°C and lose 5.18, 7.21, and 7.04 % weight respectively (Figures S2a, b, and c from their initial mass of the material, a mass loss that is attributed to the loss of physically bound water. The trough which represents the loss of structurally bound water is clearly visible for the CMS STx-1b and SWy-3 Montmorillonites (Figures S2b and c, with the last peak occurring at temperatures greater than 900 °C which is visible for all types (K10, STx-1b, and SWy-3). Interestingly up to 100°C the percent mass loss rate is approximately 1%/min and appears to follow the same path for all Montmorillonite clays, and the water weight lost was 4.9% for SWY and STX, and 4.4% for K10. This indicates that for all types of Montmorillonite investigated herein the rate of dehydration is fairly similar up to 100°, for a heat flow rate of 20°C/min, nearly the same percent of water is lost and any shrinkage due to water loss for these material could be comparative, albeit less for the K10 particles. Conversely shrinkage due to water loss is not expected or will be negligible in samples containing the calcined Kaolinite, which lost 0.4% percent weight at 100°C while temperatures were increased at 20°C/min. In terms of total mass loss, the calcined kaolinite experienced a total loss of 1.51%, while the total loss was 15.61% for STx-1b, 13.55% for SWy-3, 11.98.% for K10, and 5.3% for OPC.

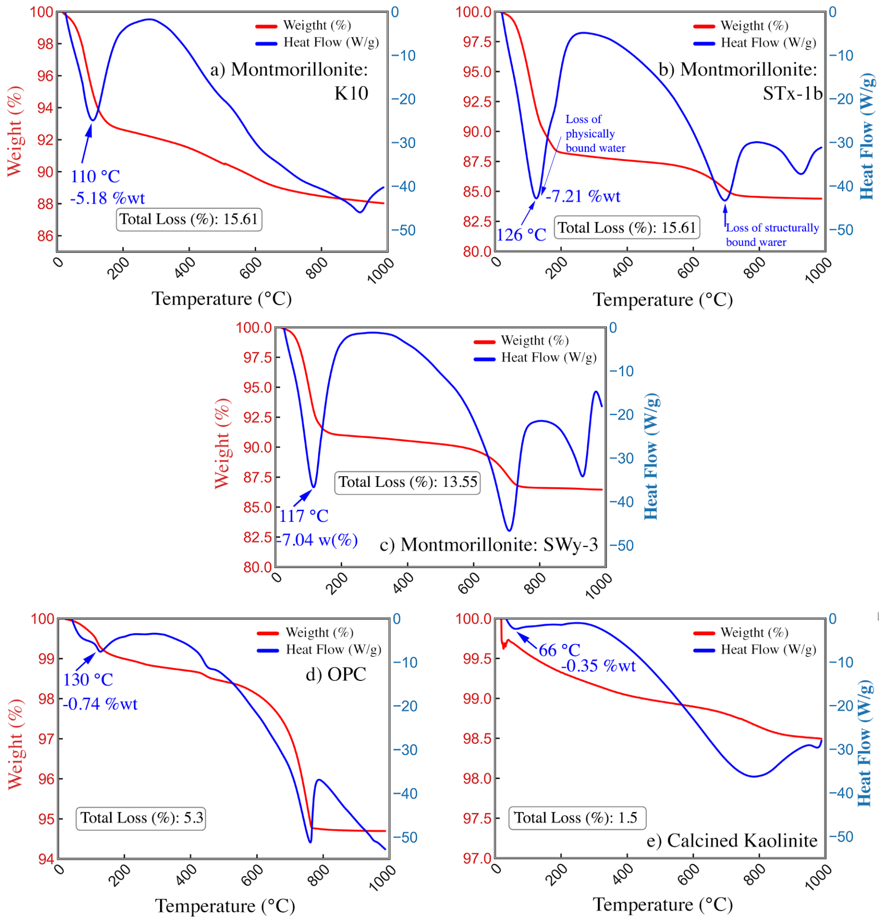

Figure S2. The percent Weight Loss of material (TGA) is plotted with the Heat Flow (DSC) at temperatures ranging from 20°C-1000°C for different Montmorillonites (a-c), OPC (d) and calcined kaolinite (e). Please note that the y-axis is not the same for all

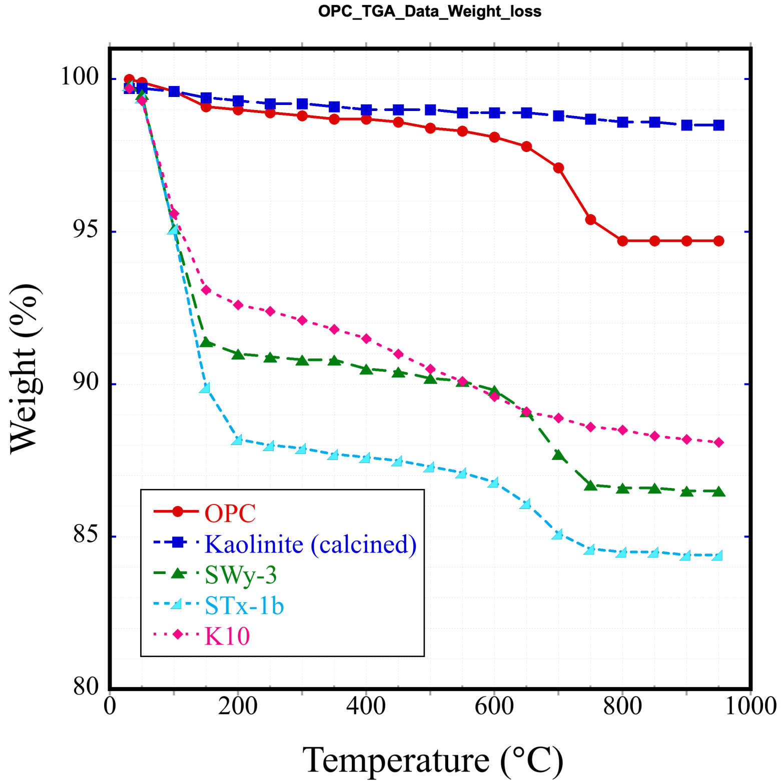

Figure S3. A comparison of the percent weight loss obtained from TGA-DSC at temperatures ranging from 20-1000°C for different Montmorillonites, OPC and, calcined kaolinite

Supplementary Note 8

Damage characterization with In-Situ X-ray Microscopy

The geo-architected rocks imaged during the loss of moisture are described above in Section Supplementary Note 2. These rocks may vary in the distribution and localization of clay material, which affects the experimentally obtained unconfined compressive strength (UCS), where the UCS of the localized clay and distributed clay specimen (CB-K10) are 3% and 60% less respectively than the UCS of mortar, which points to clay-bearing geo-architected rocks as weaker samples. To examine drying front infiltration, crack nucleation and damage propagation in the reference and clay-bearing synthetic rocks during moisture loss, experiments were conducted within a Zeiss Xradia 510 Versa X-ray Microscope on unbounded cylindrical geo-architected rock specimens. All geo-architected rock samples were imaged with a 0.4X objective, a Zeiss proprietary high energy filter (HE2), an exposure of 1.25 seconds, and at a voxel edge length resolution of approximately $\sim 40\mu m$ at the start of the monitoring period to visualize the initial state of the framework at the onset of drying. The clay-bearing (SK10) samples were intermittently re-imaged over the course of six days. All other samples were imaged at the start and end of the six day monitoring period. These moisture loss experiments were performed within the X-ray microscope system which maintains a near constant temperature of 28°C for six (6) full days.

Supplementary Note 9

Image Data Analysis for Damage Quantification and Comparison

Initial image processing and data analysis such as segmentation and visualization were performed using Object Research Systems (ORS) Dragonfly Pro software, Version 2020.2 for [Windows] software[11]. Observations regarding the condition of the microstructure (fractured or damaged vs. undamaged) were explored as this is crucial for interpretation and comparison to the PD simulated results. The different networks (unconnected pores and connected cracks) were quantified using thresholding segmentation which relied on the measured continuous grayscale that is a function of the material properties. Each pixel contains a gray shade that varies from black (0) to white (255) and the value of each pixel carries information regarding the intensity of the x–rays which can be used to extract different features and interpret the relative density of materials. To estimate the total porosity (represented by black areas in the image within the sample) with segmentation, both non-connected and connected voids with volumes greater than 0.0005 mm^3 are considered. In the 3D XRM image data, the color of locations that are representative of low density material will approach black (e.g. air voids) while the colors for high density materials tend to approach white. With this form of thresholding, features with similar gray bandwidths within the intensity histogram of the measured data are assigned into discrete groups (regions of interest, i.e. ROI) which are used to estimate the total porosity and the percent volume of cracks which consisted only of connected voids, and the extent of the zones of damage, i.e percent volume of material damaged during the advancement of the drying induced crack front.

Crack Detection and Parameter Analysis: To quantify the damage parameters for the simulated, and experimental data the Ridge Detection plugin[15] in FIJI was used. First these data are scale calibrated, color threshold segmented, binarized (converted to an image that contained only black and white), and converted to 8-bit images using FIJI[16]. All analysis were conducted on the image slices obtained at the center of the samples. For the damage (cracks) simulated with PD areas are selected based on the warmer regions in the base case image data. The ridge detection plugin[15] incorporates a modified differential geometric approach (i.e. it locates and further segments peaks and troughs in a defined image function) which is used to detect lines and their respective edges and employs Gaussian masks to estimate the derivatives of the image, which can also accommodate lines with varied widths[15]. Ridge detection explicitly models the interaction between lines, and their corresponding edges to analytically predict and remove any bias. With this plugin the position and widths (apertures) of lines of interest in an image can be captured with sub-pixel accuracy using only the first and second directional derivatives to resolve and extract the line points (ridges), and a localized search around these line points with 5 small masks to obtain the edge points[15]. Here it is used to detect the cracks (lines), to estimate the features of these entities in the analyzed image such as number of cracks (N_c), apertures, average crack length, the distribution of crack lengths, and the number of cracks per mm^2 (c_a) which is defined by the number of cracks per unit area (i.e. $c_a = N_c/mm^2$) of the sample. The ridge detector was also used to create a binary 2D crack/damage model for the simulated data for further analysis as it provides defined boundaries for cracks in the simulated data. The setting used for all samples include a high and low contrast of 230 and 87 respectively, along with $\sigma = 2.0$, and minimum and maximum line lengths = 0. For all specimens except CB-STX, upper (UT) and lower (LT) thresholds of 16.15 and 6.12 were used. While a constant σ was used to quantify and compare these data, samples CB-SWY and CB-STX would be better represented by a σ of 1.6 and 1.12 respectively. Independently generated Python scripts were used to process the data outputs from the Ridge Detection and Fraclac plugins.

Damage Distribution Analysis: To quantitatively compare damage in the distributed clay data (simulated vs. experimental)

FIJI[16] and necessary plugins are used to first process the image data as outlined in *Crack Detection and Parameter Analysis*. Then to perform morphological digital image analysis on the binarized data the FracLac plugin[17,18] is employed. All analyses were conducted on the image slices obtained at the center of the samples (simulated and experimental). The Fraclac plugin was utilized to calculate the local connected fractal dimensions (D_{LC}) which is found by using the pixel mass from concentrically placed sampling units, and the connected set at each pixel to produce a meaningful local variation in complexity[18] for the experimental and simulated 2D image data. Fraclac uses the box counting method of fractal analysis, which i) superimposes on the object of interest a grid of size η, ii) counts how many boxes, $N(\eta)$, may contain the object, and iii) repeats the task for various box sizes[16] (generating a grid of odd sized boxes for each sample investigated Figure S9). The box fractal dimension, D_F, is the negative of the slope of the line found by plotting the logarithm of $N(\eta)$ versus the logarithm of η, which provides an estimation of the change in space filling with change of observational scale[18]. Essentially, the local connected fractal dimension is a local dimension calculated for each pixel using the slope of a log-log regression line for pixel mass against box size, and is color-coded (Figure S10) to display the local variation in complexity in the image domain[18]. The mean distribution of the estimated LCFD, D_F, are compared for the complexity of the full 2D images, Figure 10 (in manuscript), of the simulated data and CB samples (SK10, STX, SWY, and SK10-5). Since the simulated distributed clay was modeled directly from the experimental CB image data, we apply the same pixel ratio obtained by calibrating the experimental CB-SK10 image with the known length of the experimental image scale bar. The comparison between the 2D center slice for the simulated-CB and experimental-CB-SK10 was expanded to examine the similarity between these data in four segments along the vertical of the 2D side view (Figure 11 in manuscript). It should be noted that the calcined Kaolinite (CB-SKAO) (Figure S4a-c) experienced no observable damage to the microstructure and damage analysis is therefore not performed. This data is only shown as a representative for a sample that was fabricated with a constituent material with a markedly different dehydration rate in comparison to the swelling clay material.

The mean distributions are compared using the two-sided Kolmogorov-Smirnov (KS) test from Python's Scipy package[19]. This is a non-parametric test that compares the underlying continuous distributions $F(x)$ and $G(x)$ of two independent samples[19]. The data (histograms of the mean distribution of complexity in the analyzed images) are prepared for analysis utilizing a well-behaved interpolation method[20] that generated curves to exactly match the given slopes of the points of the input data thus retaining a robust approximation of the original shape of the histograms. The KS-test is then applied to the interpolated data comparing the distribution for the simulated base case to all the CB experimental samples, and additionally comparing the CB-SK10 sample to the entire CB sample group. The KS-test calculated the K-statistics and p-values for a comparison, which can be used to determine characteristics of a distribution (e.g. if the K-statistics is 0 and the p-value is equal to 1 then the distribution is perfectly uniform), and the similarities in the distribution of two histograms, i.e. if two samples stem from the same distribution. Here the presumption that the sample datasets are identical such that $F(x) = G(x) forallx$ and therefore originate from the same distribution is the null hypothesis. The obtained p-value is therefore used as a measure for which this null hypothesis can either be accepted or rejected. For the data analyzed, the probability threshold that the null hypothesis is rejected, is equal to 0.05. This is a reasonable value for most applications. Consequently if the p-value is less than the threshold then the null hypothesis was rejected and it was determined that the histograms for two samples do not originate from the same distribution. From the KS test, the degree of similarity of the distribution of complexity between the PD and experimental data can be quantified, thus validating the results of the PD simulation. The readers are guided to the references for detailed information pertaining to the use of the Kolmogorov-Smirnov Test[19,21] and data interpolation[20].

Supplementary Note 10

Damage Analysis and Comparison

The rock analogues monitored under ambient laboratory conditions (ambient temperature and relative humidity) were fabricated with materials characterized by relatively similar dehydration rates for temperatures < 100°C at a heat flow rate of 20°C/min (Figure S3) and markedly different dehydration rates where little to no water is lost from the structure, e.g. calcined Kaolinite (Figures S2 & S3). The effect of using the low dehydration, non-swelling calcined kaolinite (KAO) material, which is expected to experience negligible differential shrinkage, is shown in Figure S4. Here the synthetic rock fabricated with KAO, CB-SKAO, is intact with no observable damage within the microstructure at the end of the experimental monitoring of moisture loss. Neither the map (Figure S4a nor the side (Figure S4b views show any sign of damage, and no cracks are visible at the scanning resolution of 40 μm. Furthermore, no damage in the form of cracks are visible on the 3D exterior of the sample shown in Figure S4c.

Relatively higher dehydration rates are expected from the Montmorillonite clays which all experienced > 10% total mass loss during TGA-DSC thermal analysis (Figure S3), and up to 100°C these materials were characterized by the same mass loss rate. The geo-architected rocks fabricated with these Montmorillonite clays were the only samples that experienced damage

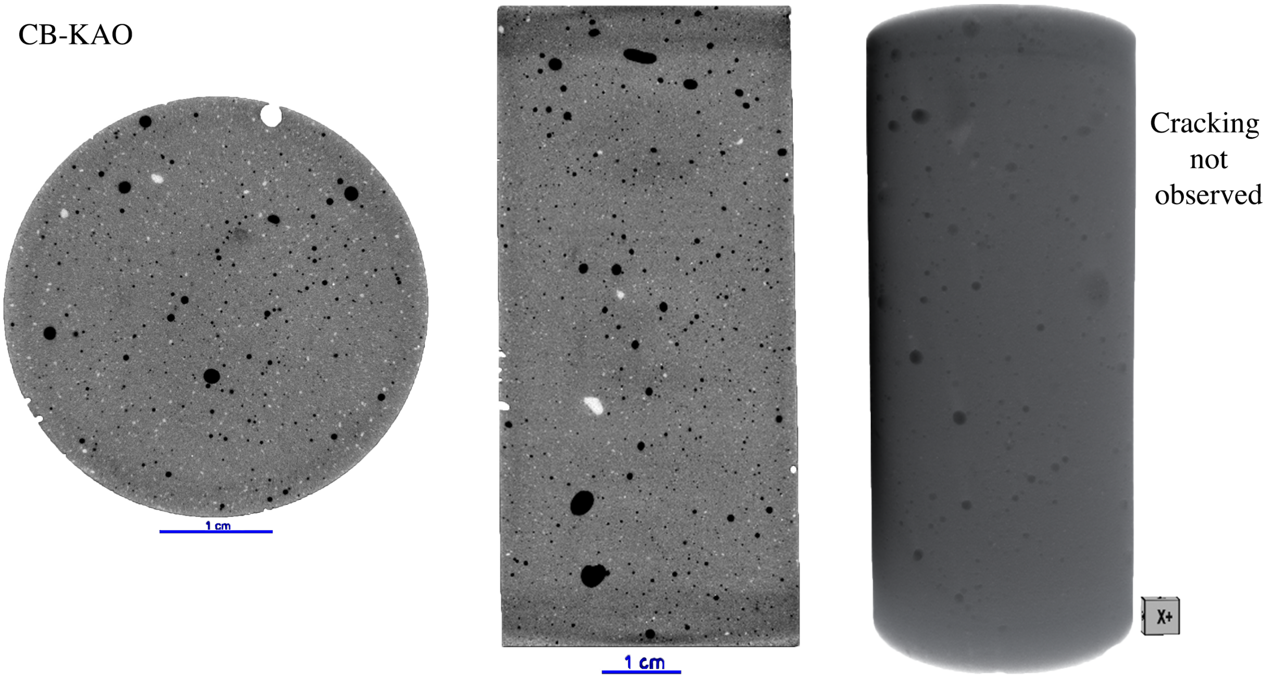

(a) Map View **(b)** 2D Side View **(c)** 3D View

Figure S4. Images of the CB-KAO distributed clay sample, fabricated with 20% calcined Kaolinite, where 2D map and side (a & b), and 3D (c) views are shown. Damage was not observed within the microstructure of this sample at the end of the moisture loss monitoring period.

within the microstructure, with cracks observable at the imaging resolution. The CB-SK10 (20% K10) and CB-STX (20% STX) synthetic rocks had the most damage to the microstructure (8.93% & 9.6% respectively) and values of c_a at the center of the sample were 18% and 13%, and 58% and 8% in the map and side views respectively. The CB-SK10 had a distinct crack network characterized by apertures in the range of $0.082 - 1.424m$ and crack lengths $0.036 - 13.78mm$, that extended from the exterior of the sample towards the center of the core. Interestingly the crack network observed in the CB-SWY sample (Figures S5, S6d, & S7d) was markedly different when compared to CB-SK10 and by extension CB-STX samples. The cracks observed within this sample are denser at the top of the core, and the existing cracks uniquely branch out from the top leaving the lower half mainly undamaged at the center of the sample. This rock analogue was fabricated with Na-montmorillonite which has the propensity to retain more water than Ca-montmorillonites, as shown in experiments performed by[22], the water demand is higher in Na-montmorillonites which have unlimited swelling capabilities and greater particle dispersion in water when compared to Ca-montmorillonites which results from the limited water absorption capabilities of the Ca-clay mineral.

Several differences in crack networks are immediately observable across the sample types (Figure 9 in the manuscript), which signals to an underlying difference in how these materials interact within the homogeneous background matrix. Cracks formed due to moisture loss and differential shrinkage of constituent materials can lead to macroscopic damage in the microstructure of rocks and quantifying these parameters may provide insight on the damage resulting from such mechanisms. For the samples in which damage is observed, the Ridge detection plugin is used to further analyze and quantify the crack features in the data to compare to the features that were also quantified for the PD image data. More information regarding this plugin can be found in Supplementary Note 9.

With Ridge Detection, cracks (lines) are successfully identified and characterized using the default algorithm and the parameters outlined in Supplementary Note 9. The overlays are shown on the binarized data (yellow and green lines) in the 2D map and side views, for samples PD simulation (Figures S6a & S7a, CB-SK10 (Figures S6b & S7b, CB-STX (Figures S6c and S7c, CB-SWY (Figures S6d & S7d, and CB-SK10-5 (Figure S7e, where only the 2D side view is shown. In addition to this these data are plotted in the box-and-whisker plots (Figure S6) & Side (S7) which provide a visual sense of the statistics for the extracted data. With Ridge Detection, apertures (crack widths), length, the number and locations of crack intersections, shape (variation in aperture, i.e. dilation or contraction along its length), and crack position was extracted. Select parameters are presented in Tables S5 and S6, along with the number of cracks per unit area per 2D image view (map and side views) for each sample type.

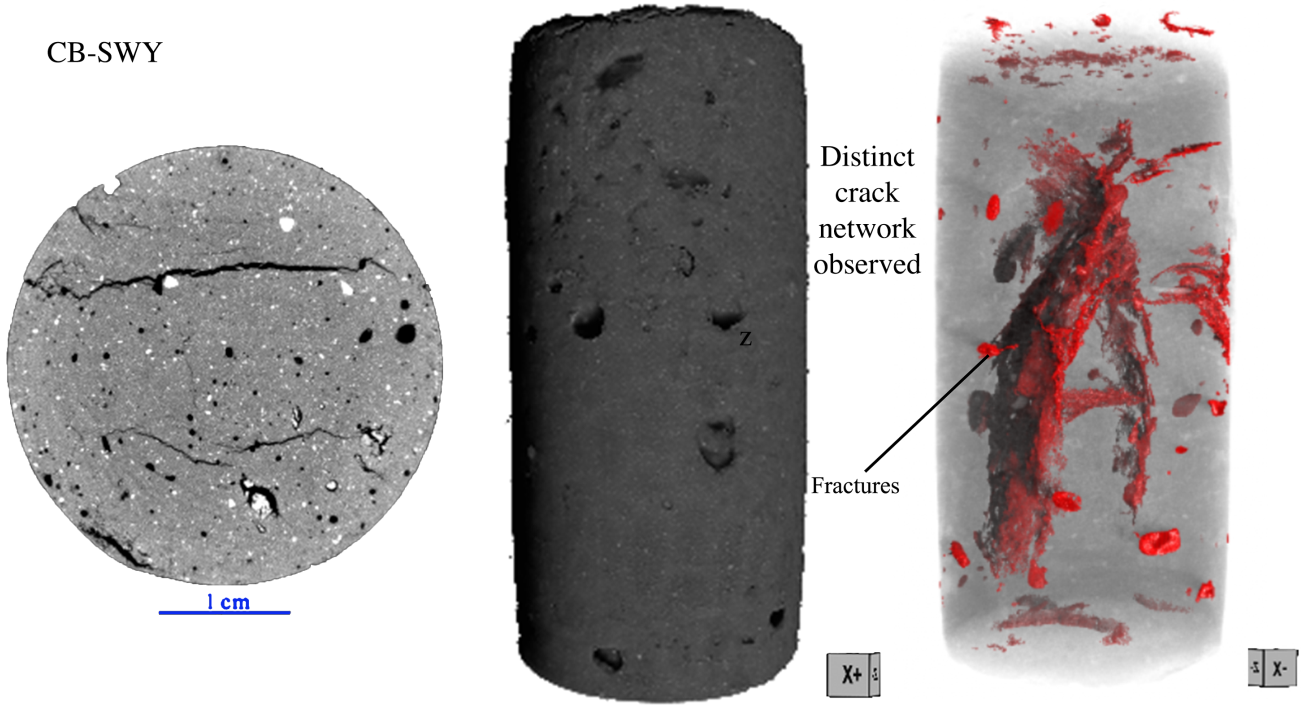

(a) Map View **(b)** 3D Exterior View **(c)** 3D Interior View

Figure S5. Images of the CB-SWY distributed clay sample, fabricated with 20% Na-Montmorillonite, where a 2D map (a) and, 3D exterior (b) and Interior (c) views are shown. Damage was observed within the microstructure of this sample, which was different from the CB-SK10 sample.

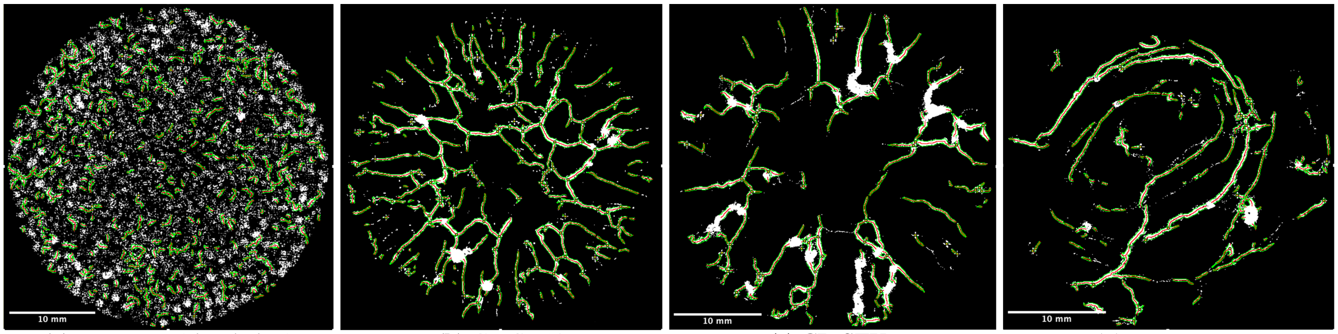

(a) Base case (simulation) $N_c = 1022$ **(b)** CB-SK10 $N_c = 197$ **(c)** CB-STX $N_c = 601$ **(d)** CB-SWY $N_c = 88$

Figure S6. 2D Binarized map view images for the (a) peridynamic model, and experimental samples (b) CB-SK10, (c) CB-STX, and (d) CB-SWY. The map view image data for CB-SK10-5 is not shown as only one small crack is observed. Crack lengths are highlighted by the red lines (ridges along the length) and apertures (crack widths) by the green lines which were detected using the Ridge Detection plugin[15]

The average crack lengths extracted from the center slice for each sample type for both the map (Figure S8a) and side views (Figure S8b), along with the average apertures (Figures S8c and S8d), and average maximum aperture values (Figures S8e and S8f) show that most of the features are larger in the map view. This could be related to the fact that all samples are unbounded and free to expand without being hindered by an external confinement or force. Crack parameters for the PD data, excluding the crack length, correspond well to the values obtained for the experimental samples in the 2D side view. In comparison to the CB-SK10 sample, the average apertures extracted from the PD binarized cracks were 18% wider than the average aperture for

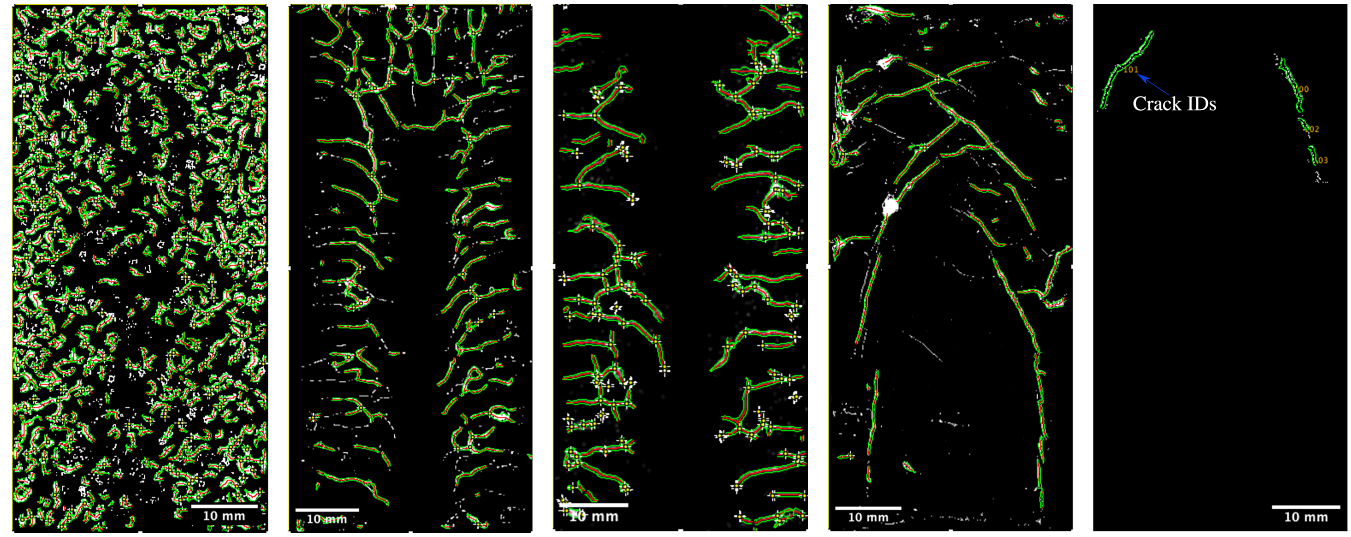

(a) Base case (simulation) $N_c = 2135$ **(b)** CB-SK10 $N_c = 384$ **(c)** CB-STX $N_c = 232$ **(d)** CB-SWY $N_c = 165$ **(e)** CB-SK10-5 $N_c = 13$

Figure S7. 2D Binarized side view images for the (a) peridynamic model, and experimental samples (b) CB-SK10, (c) CB-STX, and (d) CB-SWY. The map view image data for CB-SK10-5 is not shown as only one small crack is observed. Crack lengths (red lines are the ridges of the cracks along the length), and apertures (marked by edges used to define crack widths - green lines) that were detected using the Ridge Detection plugin[15]

	Simulated	CB-SK10	CB-STX	CB-SWY	CB-SK10-5
c_a (%)	98	18	58	8	< 1
N_c	1022	197	601	88	4
% of Jointed cracks	65	52	93	69	0
**Mean Values (mm)**					
Length	1.446	2.712	1.001	3.462	7.286
Aperture	0.562	0.459	0.434	2.136	0.511
Minimum Aperture	0.346	0.254	0.316	0.952	0.053
Maximum Aperture	0.821	0.708	0.554	3.196	0.768
**Range (mm)**					
Minimum Aperture	0.160	0.090	0.008	0.000	0.021
Maximum Aperture	1.375	1.424	1.222	4.665	1.040
Minimum Length	0.057	0.080	0.069	0.085	2.327
Maximum Length	8.858	13.777	10.798	16.905	14.373

Table S5. Parameters extracted from 2D Map view image data from crack analysis with the ridge detection plugin[15], in FIJI[16]

CB-SK10, and the average crack length for the PD base case was 47% less than the crack length extracted from the map view of the experimental image data. In the side view with the exception of the crack length, the additional crack parameters for both the PD base case and CB-SK10 are very close, e.g. the average aperture, minimum, and maximum aperture values of the base case are 97%, 102%, and 89% of the CB-SK10 values. If the simulated data are compared to the damage network formed in sample CB-SWY, which is visibly different from that of the CB-SK10 sample, the crack length is 58% shorter, the average crack aperture is 74% thinner, and the minimum and maximum crack apertures are thinner by 64% and 74% respectively. For the experimental samples a higher N_c (601 cracks with 93% joints) was observed in the map view for the CB-STX rock analogue, and in the side view the N_c for CB-K10 was higher (384 cracks with 80% jointed). In fact for all samples the average minimum

	Simulated	CB-SK10	CB-STX	CB-SWY	CB-SK10-5
c_a (%)	74	13	8	6	< 1
N_c	2135	384	232	165	13
% of jointed cracks	84	80	80	52	69
**Mean Values (mm)**					
Length	0.477	1.244	1.217	1.497	0.929
Aperture	0.263	0.270	0.293	0.249	0.232
Minimum Aperture	0.175	0.171	0.192	0.154	0.170
Maximum Aperture	0.364	0.407	0.434	0.370	0.313
**Range (mm)**					
Minimum Aperture	0.050	0.082	0.092	0.073	0.046
Maximum Aperture	0.763	0.744	0.738	0.684	0.487
Minimum Length	0.037	0.036	0.042	0.055	0.062
Maximum Length	3.691	7.000	6.967	12.579	3.696

Table S6. Parameters extracted from 2D Side view image data analyzed with the ridge detection plugin[15], in FIJI[16]

and maximum apertures were larger in the map view. In both the map and side view analyses, longer cracks were observed for the synthetic rock fabricated with Na-Montmorillonite, CB-SWY, with a maximum detected length of 16.9 mm. The shape of the cracks, and the extent of the crack network in this sample was different from other experimental samples and the simulated data. The highest value of N_c was extracted from the binarized image for the PD data. The crack parameters, such as aperture and lengths however were comparable to the the experimental data, especially in the 2D image side view. Box-and-whisker plots show the spread of these data (Figure S8), and display the degree of dispersion, skewness of the data, minimum, maximum, mean, and quartile range values. The lack of cracks in CB-SKAO indicate that if the differential shrinkage rate is less than or comparable to that of the material used in the background homogeneous medium damage to the microstructure may not be seen.

To extract a robust description of crack network complexity, the Fraclac plugin is used which executes a fractal analysis box counting method to examine the variations in crack density within the image space, and color-code the binarized image data in relation to the variation in the locally connected fractal dimension D_{LC} over the image space. The local connected fractal dimension analysis is performed using boxes of odd sizes. Box counting is the main data gathering method for the Fraclac plugin, and this grid consisting of boxes of different sampling sizes is shown in Figure S9 for the different sample types. The box counting method is a process where boxes containing foreground pixels and the number of pixels per box are estimated and in turn used to determine the fractal dimension. Details on the LCFD scanning method can be found in Supplementary Note 9.

The resulting images (Figures S10a-e) are the basis for the fractal dimensions obtained using the LCFD scanning method. The values color-coded onto the binary data are representatives of D_{LC} which is computed for each pixel via a method equivalent to how D_F is estimated that incorporates the pixel mass from concentrically placed sampling units and the connected set located at each pixel[17,18]. These values produce a map of complexity (Figure S10) and local variation at pixel resolution. This distribution map for the PD data show higher values of D_{LC} concentrated around the exterior of the PD model, which demonstrates that the damage is higher in these areas, i.e. more foreground pixels fill the boxes of the grid. Such high damage areas are more displaced in the experimental distribution maps of D_{LC}. The fractal dimension, D_F which represent the mean distribution of the complexity per sample, is an index of how this complexity and details in an image change with resolution. This mean distribution of D_F is plotted for all samples in Figure 10 and for segmented portions of the PD data in comparison to the experimental model CB-SK10 in Figure 11.

The KS-test is used to analyze the similarities between the mean distribution of the PD data and the histograms for the experimental samples, the experimental mean distribution amongst the samples, and for segments of the PD data and the experimental CB-SK10 sample to get a clear sense of how the damage (complexity) may change along the vertical of the 2D PD versus experimental data, the histograms were plotted together in Figure S11. The similarities between these distributions for the base case of the PD model versus the experimental data, for samples CB-SK10, STX, SWY, and SK10-5 are shown in Figures 10 and 11 (in manuscript). The mean of the histograms are approximately the same (Table 1 in the main manuscript) and quantification of similarities in the distribution of the PD data versus the CB samples reveal similarities in the mean

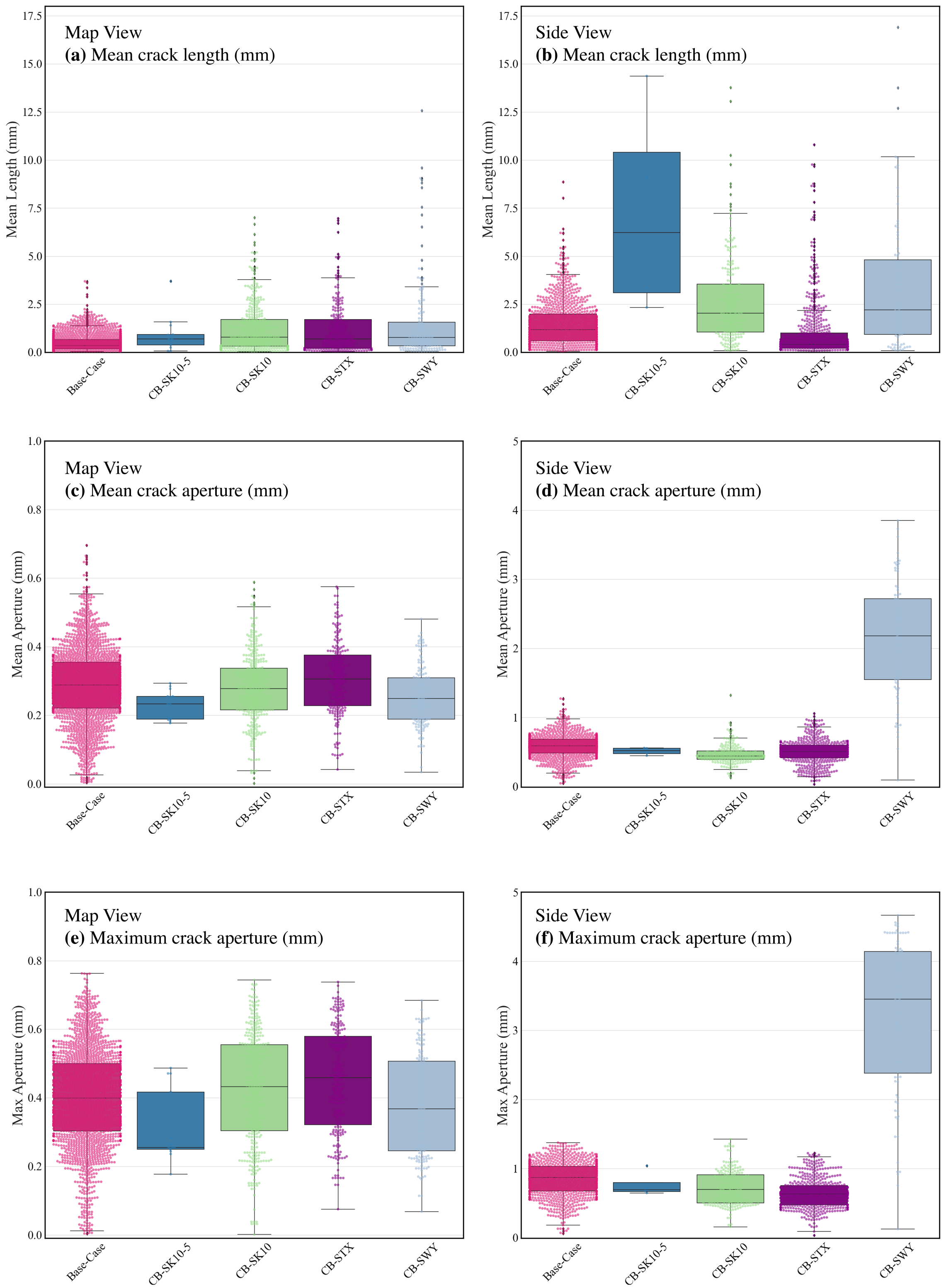

Figure S8. Statistics of crack parameters are presented with box-and-whisker plots for crack distributions extracted from the 2D Map and Side view image for the i) average crack length (Figures (a) & (b)), ii) the spread of average aperture (width) per sample type (Figures (c) & (d), and iii) the average of maximum apertures for each segmented crack (Figures (e) & (d)) extracted from the Base case, CB-SK10-5, CB-SK10, CB-SWY, and CB-STX samples.

distribution of damage for all samples except CB-SK10-5. For the comparison between segments of the PD base case verse the CB-SK10 sample, similarities in the distribution exist for all segments except the fourth, which is the last segment of the sample (Figure 11 in manuscript). Furthermore based on the p-values extracted (CB-STX=0.40, CB-SWY = 0.47. and CB-SK10-5 = 0.08) for the comparison of the CB-SK10 sample to the other experimental data, it was found that all mean distribution of damage appear to arise from similar distributions as the CB-SK10 sample. If the same comparison is performed for the CB-STX sample, only CB-SK10 (p-value=0.4) and CB-SWY (p-value = 0.19) bear any similarity in distribution.

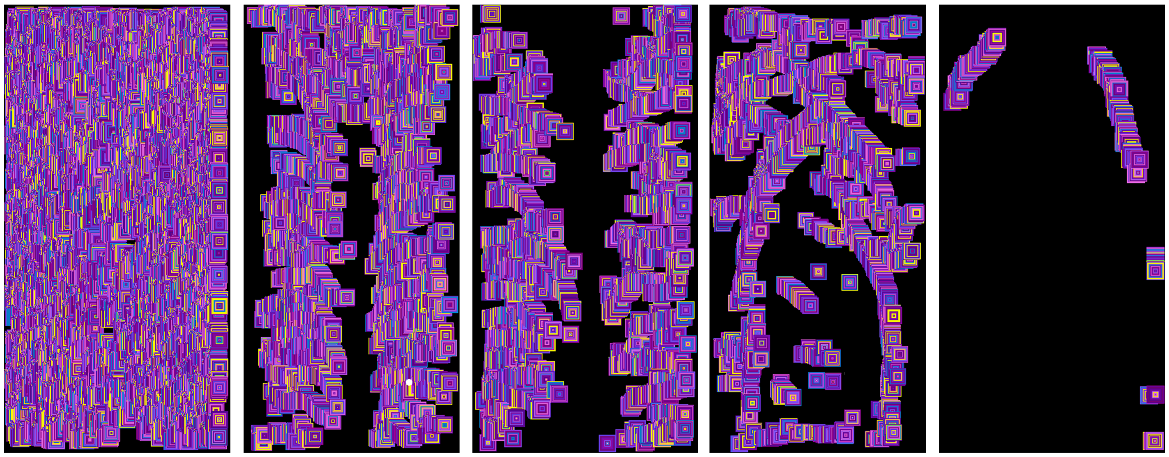

(a) Base case (simulation) **(b)** CB-SK10 **(c)** CB-STX **(d)** CB-SWY **(e)** CB-SK10-5

Figure S9. Grids of odd sized boxes per each image for LCFD analysis where (a) is the peridynamical model, and the analogue rocks (b) CB-SK10, (c) CB-STX, (d) CB-SWY, and (e) CB-SK10-5 fabricated with 5% percent Montmorillonite

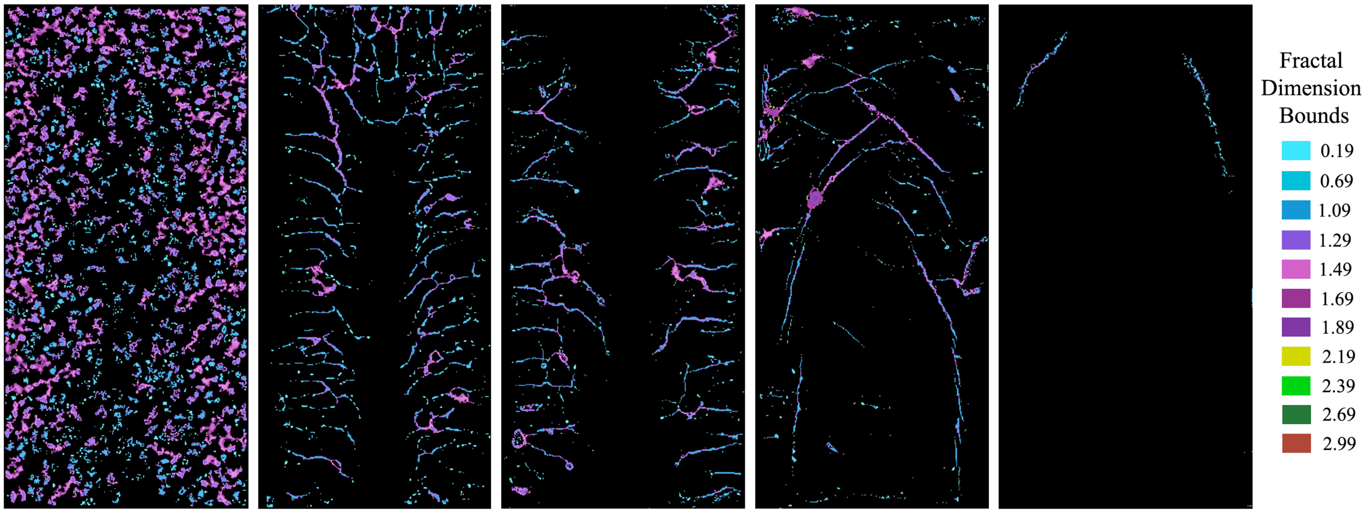

(a) Base case (simulation) **(b)** CB-SK10 **(c)** CB-STX **(d)** CB-SWY **(e)** CB-SK10-5

Figure S10. Connected sets color-coded according to the Local Connected Fractal Dimension (LCFD) at each foreground pixel for (a) the peridynamical model, and analogue rocks (b) CB-SK10, (c) CB-STX, (d) CB-SWY, and (e) CB-SK10-5

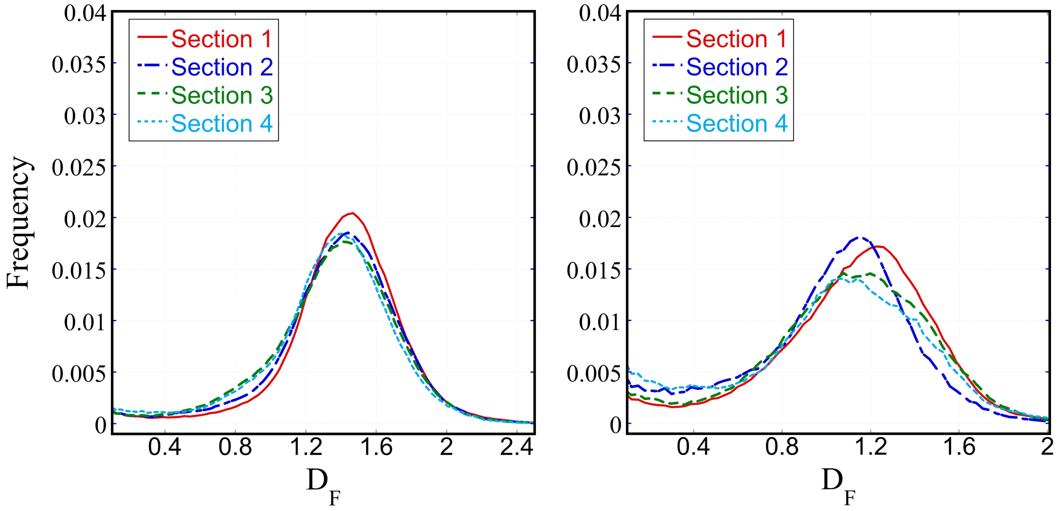

(a) PD Base case (D_F per vertical segment) **(b)** CB-SK10 (D_F per vertical segment)

Figure S11. The mean distribution of D_F for each segment of the (a) PD base case and (b) experimental data for sample CB-SK10 as shown in Figure 11 (in manuscript)

Supplementary Note 11

Drying Model

The diffusion equation

$$\frac{\partial q}{\partial t}(\mathbf{x},t) = D_c \Delta q(\mathbf{x},t). \tag{1}$$

may be employed to simulate drying[23], where D_c is the classic diffusivity constant and Δ is the Laplacian operator. In this formulation, the $q(\mathbf{x},t)$ and D_c refer to the moisture content and moisture diffusivity respectively. In the present work we mimic this classical formulation; however, diffusion is simulated with a non-local formulation of the model:

$$\frac{\partial q}{\partial t}(\mathbf{x},t) = D_p \int_{\mathscr{H}_\mathbf{x}} \lambda(\mathbf{x}'-\mathbf{x})\left(q(\mathbf{x}',t) - q(\mathbf{x},t)\right) d\mathbf{x}'. \tag{2}$$

The set $\mathscr{H}_\mathbf{x}$ is the neighborhood of $\mathbf{x}$, i.e., the material points $\mathbf{x}$ interacts with directly, which we set to $\mathscr{H}_\mathbf{x} := B_\delta(\mathbf{x})$ the ball of radius δ about $\mathbf{x}$. The function $\lambda(\mathbf{x}'-\mathbf{x})$ controls the degree of interaction between material points $\mathbf{x}$ and $\mathbf{x}'$, and frequently in the literature a radially decaying function is employed. In this work we utilize the inverse distance function

$$\lambda(\mathbf{x}'-\mathbf{x}) = \frac{1}{\|\mathbf{x}'-\mathbf{x}\|}. \tag{3}$$

In order for (1) and (2) to agree up to fourth-order terms, the peridynamic diffusivity constant D_p is related to the classical diffusivity constant D_c through the relation:

$$D_p = \frac{6D_c}{\pi\delta^4}. \tag{4}$$

Supplementary Note 12

Mechanics Model

Similar to the reformulation of the classical diffusion equation, in bond based peridynamics the integro-differential reformulation of the balance of momentum

$$\rho(\mathbf{x})\ddot{\mathbf{u}}(\mathbf{x},t) = \int_{\mathscr{H}_\mathbf{x}} \mathbf{f}(\mathbf{u}(\mathbf{x}',t),\mathbf{u}(\mathbf{x},t),\mathbf{x}',\mathbf{x}) dV_{\mathbf{x}'}, \tag{5}$$

is the governing equation of motion. Here $\rho(\mathbf{x})$ is the mass density at material point $\mathbf{x}$, $\ddot{\mathbf{u}}$ is the second derivative in time of the displacement field $\mathbf{u}$, $\mathscr{H}_x$ is the neighborhood of $\mathbf{x}$, and $\mathbf{f}$ is the pairwise force function describing the influence material point $\mathbf{x}$' exerts on material point $\mathbf{x}$. For convenience we employ a shorthand:

$$\xi := \mathbf{x}' - \mathbf{x} \quad \text{and } \eta := \mathbf{u}(\mathbf{x}',t) - \mathbf{u}(\mathbf{x},t). \tag{6}$$

In this work we modify the prototype microelastic brittle (PMB) model[24] where the force function $\mathbf{f}$ is described by

$$\mathbf{f}(\mathbf{u}(\mathbf{x}',t),\mathbf{u}(\mathbf{x},t),\mathbf{x}',\mathbf{x}) := cs(t,\eta,\xi)\mu(t,\xi)\frac{\eta+\xi}{|\eta+\xi|}, \tag{7}$$

where c is a material dependent constant,

$$s(t,\eta,\xi) := \frac{|\eta+\xi|-|\xi|}{|\xi|}, \tag{8}$$

is the stretch in the bond between $\mathbf{x}$ and $\mathbf{x}'$, and

$$\mu(t,\xi) := \begin{cases} 1, & \text{if } s(t',\eta,\xi) < s_0 \quad \forall t' \le t \\ 0, & \text{else} \end{cases} \tag{9}$$

is a history-dependent binary-valued function the describes whether a bond is intact or broken based on a critical stretch s_0. The material parameters c and s_0 are material dependent, and for convenience they are typically related to commonly measured material parameters. The parameter c in Equation (7) is typically associated with an elastic constant; however, in classical linear elasticity the model is described by two engineering constants such as Poisson's ratio ν and the bulk modulus K. Due to the use of a pairwise force function in bond-based peridynamics, only materials nearly satisfying Cauchy's relations are able to be described[25]. In the case of isotropy, Cauchy's relations reduce to a fixed Poisson's ratio[26] of $\frac{1}{4}$. The parameter c is then related to the bulk modulus K through the relation:

$$c = \frac{18K}{\pi\delta^4}, \tag{10}$$

in which case (5) agrees with the classical linear elastic equation of motion up to fourth order terms. The critical stretch s_c may be related to the fracture energy G_o through the relation[24]:

$$s_c = \sqrt{\frac{5G_o}{8K\delta}}. \tag{11}$$

Damage within the peridynamics framework is described by broken bonds; however, local nodal properties are simpler to deal with, and so it is common to describe the damage at a node $\mathbf{x}$ by[24]

$$\phi(\mathbf{x},t) = 1 - \frac{\int_{\mathscr{H}_\mathbf{x}} \mu(t,\xi)dV_{\mathbf{x}'}}{\int_{\mathscr{H}_\mathbf{x}} dV_{\mathbf{x}'}}. \tag{12}$$

In order to implement a dependence on drying into the mechanical model, a dependence on the moisture content $q(\mathbf{x},t)$ is incorporated into the PMB model (7). For convenience, we normalize the moisture variable in (2) so that $q(\mathbf{x},t) \in [0,1]$, where $q(\mathbf{x},t) = 1$ describes a material point that is fully saturated and $q(\mathbf{x},t) = 0$ describes a material point which is devoid of moisture. Moisture dependence is implemented into the PMB model (7) by adding a dependence on moisture to the stretch s, critical stretch s_c, and peridynamic elastic parameter c. For simplicity, the material parameters c and s_c are assumed to have a linear relationship with the moisture content parameter $q(\mathbf{x},t)$:

$$\begin{aligned} c(t,\mathbf{x}) &= c + (1-q(\mathbf{x},t))\Delta c, \\ s_c(\mathbf{x},t) &= s_c + (1-q(\mathbf{x},t))\Delta s_c, \end{aligned} \tag{13}$$

where c and s_c correspond to the material parameters in the wet material while Δc and Δs_c correspond to the difference in material properties in dry ($q(\mathbf{x},t) = 0$) and wet ($q(\mathbf{x},t) = 1$) materials. Shrinkage is incorporated into the PMB model by introducing a parameter $\alpha \in (0,1]$ into the equation for bond stretch[27]:

$$s(\eta,\xi) = \frac{\|\eta+\xi\| - \theta(\alpha,q(\mathbf{x},t))\|\xi\|}{\theta(\alpha,q(\mathbf{x},t))\|\xi\|}, \tag{14}$$

where

$$\theta(\alpha, q(\mathbf{x},t)) := 1 - (1 - q(\mathbf{x},t))\alpha. \tag{15}$$

When $\alpha < 1$ the stress-free reference volume is reduced, inducing shrinkage.

The coupling employed in our model is a weak coupling in that the moisture content in the cement affects the mechanics, but the mechanics do not have an effect on the drying in the material. While this is a significant simplification, several characteristics of the crack networks appearing in experimental results are captured.

Supplementary Note 13

Note 13.1 Numerical Implementation

The coupling between the drying model (2) and the mechanics model (5) is assumed to be weak, in that the moisture content $q(\mathbf{x},t)$ is independent of the displacement $\mathbf{u}(\mathbf{x},t)$. The moisture content is solved for by employing a backward Euler method:

$$q^{n+1}(\mathbf{x}_i) - q^n(\mathbf{x}_i) = \Delta t \sum_{\mathbf{x}_j \in \mathcal{N}(\mathbf{x}_i)} \lambda(\mathbf{x}_j - \mathbf{x}_i)(q^{n+1}(\mathbf{x}_j) - q^{n+1}(\mathbf{x}_i))V_{\mathbf{x}_j}. \tag{16}$$

Here Δt is the change in time from time step n to step $n+1$, the superscripts signify evaluation at time step n or $n+1$, $\mathcal{N}(\mathbf{x}_i)$ is the discrete neighborhood of $\mathbf{x}_i$, and $V_{\mathbf{x}_j}$ is the volume corresponding to material point $\mathbf{x}_j$. The moisture content $q(\mathbf{x},t)$ is used as a loading condition for the mechanics model through the reformulated stretch in equation (14). While there have been some attempts to include damage into implicit solvers in peridynamics (e.g.), damage still poses several challenges to obtaining a true quasi-static solution. The method employed in this work to obtain an approximation to a quasi-static solution is dynamic relaxation[28–30]. In this method a damping term is introduced in a discrete formulation of (5):

$$\rho(\mathbf{x}_i)\ddot{\mathbf{u}}(\mathbf{x}_i,t) - \gamma\dot{\mathbf{u}}(\mathbf{x}_i,t) = \sum_{\mathbf{x}_j \in \mathcal{N}(\mathbf{x}_i)} \mathbf{f}(\mathbf{u}(\mathbf{x}_j,t), \mathbf{u}(\mathbf{x}_i,t), \mathbf{x}_j, \mathbf{x}_i) dV_{\mathbf{x}_j} + \mathbf{b}(\mathbf{x}_i,t), \tag{17}$$

Equation (17) is then solved with a Velocity-Verlet scheme:

$$\begin{cases} \dot{\mathbf{u}}^{n+\frac{1}{2}} = \left(1 - \frac{\gamma\Delta t}{2\delta}\right)\dot{\mathbf{u}}^n + \frac{\Delta t}{2}\ddot{\mathbf{u}}^n \\ \mathbf{u}^{n+1} = \mathbf{u}^n + \Delta t\dot{\mathbf{u}}^{n+\frac{1}{2}} \\ \text{Calculate } \mathbf{F}^{n+1} \\ \dot{\mathbf{u}}^{n+1} = \left(\frac{1+\gamma\Delta t}{2\rho}\right)^{-1}\left(\dot{\mathbf{u}}^{n+\frac{1}{2}} + \frac{\Delta t}{2\rho}\mathbf{F}^{n+1}\right) \end{cases}$$

where $\mathbf{F}^{n+1}$ corresponds to the right-hand side of (17) at step $(n+1)$.

Supplementary Note 14

Numerical Parameters

The simulations require several material properties such as the shrinkage, bulk modulus, moisture diffusivity, and energy release rates. The shrinkage properties of the mortar and clay components of the composites need to be estimated. Here, the mortar is treated as a homogeneous material, and we have used variation in the properties reported for ordinary Portland cement (OPC) as the base material. It has been reported that the addition of inert fillers does not alter the shrinkage properties of cement paste[31], so significant variation when comparing experimental and computational results is not expected. Measurable shrinkage of OPC primarily occurs during hydration[32], dehydration at temperatures above 100°C[33,34], and degradation[35]. Under these conditions there are significant compositional changes, while at lower temperatures there are limited changes in the OPC structure and only unbound water is removed via dehydration[36,37]. For this study it is assumed that most of the water loss and shrinkage occurs in the clay portion of the models. Since there is negligible shrinkage of the mortar, it is assumed that the elastic and fracture properties of the OPC are also unchanged during the drying process. For hardened cement paste (W/C = 0.87) the fracture toughness (K_{IC}) is 2.8 MPa$\sqrt{\text{m}}$, the shear modulus (G) is 12.0 GPa, and K is 20.0 GPa.[27]

The clay used in this study, montmorillonite, can shrink or swell based on the presence of water between the clay sheets. At temperatures below 120°C water loss is from water trapped between the clay sheets, with phase transformations occurring at temperatures above 120°C[38]. The d-spacing of the basal plane for montmorillonite can expand through a range of ∼19Å to

~9Å[39], which relates to a maximum shrinkage of ~50% of the original volume. To correlate with experimental observations, a more realistic range is selected which sees a decrease in the d-spacing from 15Å to 12Å, this range is consistent with a decrease from two layers of water molecules between the clay sheets to one layer[39]. Along with the change in volume, and consequently density, the removal of trapped water also alters the elastic properties of the clay since hydrogen bonds stabilize the basal plane. Experimental work by Vanorio, Prasad and Nur[40] using atomic force acoustic microscopy reported K values between 6-12 GPa and S values between 4-6 GPa for kaolinite, montmorillonite, and smectite clay minerals. The reported K value of Na-montmorillonite is ~11 GPa and S value is ~5.5 GPa. Findings from molecular dynamic simulation data of the mechanical properties of Na-montmorillonite by Ebrahimi et al[39], indicate that a decrease in d-spacing from 15Å to 12Å increases the elastic constants by 15-20%. Therefore, a similar increase in the elastic properties of clay during shrinkage is anticipated, and 12.7 GPa is used for the K input and 6.6 GPa is used for S. While previous discussion focused on changes in K and elastic modulus (E) of clay in OPC, the K_{IC} is also required to calculate the critial stretch parameter s_c in (13). Values reported from flexural tests of clay soil[41], kaolinite[42], and Iro and Ewuya clay[43], estimate K_{IC} between 0.01-0.24 MPa$\sqrt{\text{m}}$ for clays. Here an average value of these studies is used, with the hydrated clay having a K_{IC} of 0.08 MPa$\sqrt{\text{m}}$. Changes in fracture properties with dehydration are much more difficult to identify due to variations in response of the different clays. Wang et al.[41] identified that K_{IC} increases from 0.05 to 0.10 MPa$\sqrt{\text{m}}$ when the water content in clay soil decreases from 20% to 16%. Thus, a K_{IC} of 0.18 MPa$\sqrt{\text{m}}$ was used for the dehydrated clay samples. The fracture and mechanical properties for the OPC and clay (hydrated and dehydrated) used in this study are included in Table S8.

K_{IC} and K are used to calculate the s_c according to the relationship described by Silling and Askari[24] based on the strain energy release rate (G) and the horizon (δ):

$$s_c = \sqrt{\frac{5G}{9K\delta}} \tag{18}$$

G for Mode I conditions can be calculated from K_{IC} and E assuming Poisson's ratio is equal to 0.25 by the equation:

$$G = \frac{{K_{IC}}^2}{E} \tag{19}$$

The critical stretch s_C is a function of the horizon δ used in the simulation. In our simulation we selected a horizon three times the mesh spacing, which is frequently employed in peridynamic simulations[44,45]. The bulk modulus K and critical stretch s_c used in the simulations are included in Table S8.

Bond Material	Mortar-Mortar	Mortar-Air	Mortar-Clay	Air-Air	Air-Clay	Clay-Clay
D_c	1.0E-3	5.0E-2	2.0E-2	5.0E-2	5.0E-2	5.0E-2

Table S7. Diffusivity parameters for the numerical experiments. Air refers to the fictitious zone around the cylinder which is utilized to simulate Robin boundary conditions

Bond Material	Mortar-Mortar	Mortar-Clay	Clay-Clay
Bulk modulus (K (MPa))	2.00E4	1.55E4	1.10E4
Critical stretch (s_c)	2.00E-3	1.00E-3	1.85E-4
Shrinkage (α)	1.00E-3	1.00E-2	2.00E-1
Change in bulk modulus (ΔK (MPa))	0.00E0	0.00E0	1.70E3
Change in critical stretch (Δs_c)	0.00E0	0.00E0	1.30E-4

Table S8. Mechanics parameters for the numerical experiments including bulk modulus K, critical stretch s_c, shrinkage parameter α, change in the bulk modulus ΔK, and change in the critical stretch Δs_c when the mortar and clay is dehydrated.

Supplementary Note 15

Drying Rates

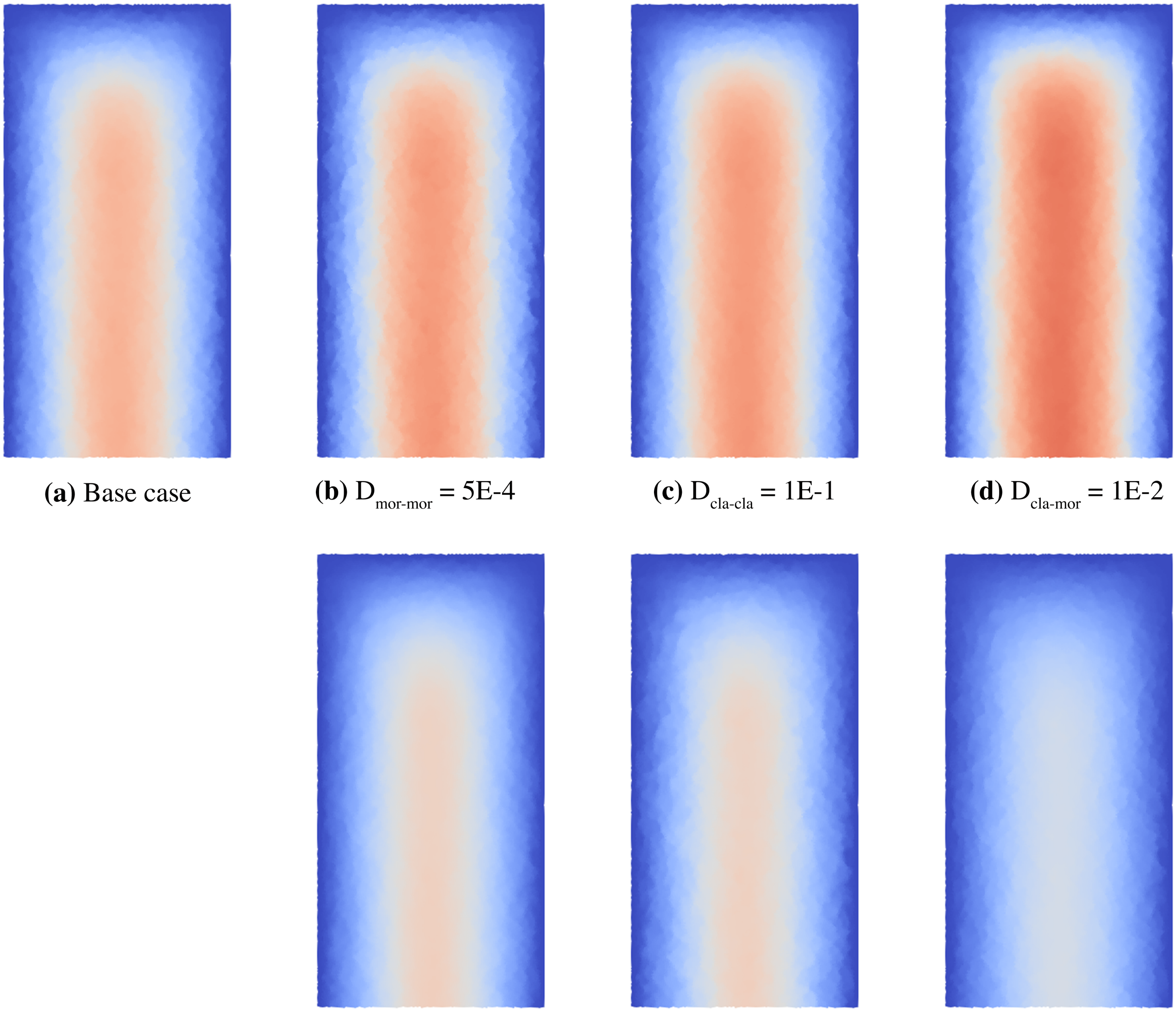

(a) Base case **(b)** $D_{mor\text{-}mor} = 5E\text{-}4$ **(c)** $D_{cla\text{-}cla} = 1E\text{-}1$ **(d)** $D_{cla\text{-}mor} = 1E\text{-}2$

(e) $D_{mor\text{-}mor} = 2E\text{-}3$ **(f)** $D_{cla\text{-}cla} = 4E\text{-}1$ **(g)** $D_{cla\text{-}mor} = 4E\text{-}2$

Figure S12. Final moisture profile in plane $x = 0$ for various diffusivity constants.

Acknowledgements

This work was funded by the Laboratory Directed Research and Development (LDRD) program of Sandia National Laboratories. Sandia National Laboratories is a multi-mission laboratory managed and operated by National Technology and Engineering Solutions of Sandia, LLC., a wholly owned subsidiary of Honeywell International, Inc., for the U.S. Department of Energy's National Nuclear Security Administration under contract DE-NA0003525. This paper describes objective technical results and analysis. Any subjective views or opinions that might be expressed in the paper do not necessarily represent the views of the U.S. Department of Energy or the United States Government. L.J.P.N. and C.M. acknowledge support of the experimental portion of this work by the U.S. Department of Energy, Office of Science, Office of Basic Energy Sciences, Geosciences Research Program under Award Number (DE-FG02-09ER16022). This work was supported by the U.S. Department of Energy Office of Nuclear Energy, through the Office of Spent Fuel and Waste Science and Technology (SFWST) Research and Development Campaign (DOE NE- 81) within the Office of Spent Fuel and Waste Disposition.

References

1. U.S.-Silica. Sil-co-sil: Bright white, high-purity ground silica (2019).

2. LafargeHolcim:North-America:Inc. Lafarge portland cement (cement): Safety data sheet (2015).

3. Sigma-Aldrich. Specification sheet.

4. Clay Mineral Socieity, Olphena, H. V. & Fripiat, J. J. Physical and Chemical Data of Source Clays.

5. Mermut, A. R. & Cano, A. F. Baseline studies of the clay minerals society source clays: chemical analyses of major elements. *Clays Clay Miner.* **49**, 381–386 (2001).

6. Marty, N. C., Tournassat, C., Burnol, A., Giffaut, E. & Gaucher, E. C. Influence of reaction kinetics and mesh refinement on the numerical modelling of concrete/clay interactions. *J. Hydrol.* **364**, 58–72 (2009).

7. Bergaya, F. & Lagaly, G. *Handbook of clay science* (Newnes, 2013).

8. Teich-McGoldrick, S. L., Greathouse, J. A., Jove-Colon, C. F. & Cygan, R. T. Swelling properties of montmorillonite and beidellite clay minerals from molecular simulation: comparison of temperature, interlayer cation, and charge location effects. *The J. Phys. Chem. C* **119**, 20880–20891 (2015).

9. Artioli, G. & Bullard, J. W. Cement hydration: the role of adsorption and crystal growth. *Cryst. Res. Technol.* **48**, 903–918 (2013).

10. Shackley, M. S. *X-Ray Fluorescence Spectrometry (XRF) in Geoarchaeology* (Springer-Verlag New York, 2011), 1 edn.

11. Inc., O. R. S. Dragonfly pro software, version 2020.2 for [windows] from object research systems (ors) inc. montreal, canada (2020). [Computer software].

12. Grim, R. E. & Rowland, R. A. Differential thermal analysis of clay minerals and other hydrous materials. part 1. *Am. Mineral. J. Earth Planet. Mater.* **27**, 746–761 (1942).

13. Grim, R. E. & Rowland, R. A. Differential thermal analysis of clays and shales, a control and prospecting method. *J. Am. Ceram. Soc.* **27**, 65–76 (1944).

14. Lothenbach, B., Durdzinski, P. & De Weerdt, K. Thermogravimetric analysis. *A practical guide to microstructural analysis cementitious materials* **1** (2016).

15. Steger, C. An unbiased detector of curvilinear structures. *IEEE Transactions on pattern analysis machine intelligence* **20**, 113–125 (1998).

16. Schindelin, J. *et al.* Fiji: an open-source platform for biological-image analysis. *Nat. methods* **9**, 676–682 (2012).

17. Landini, G., Murray, P. I. & Misson, G. P. Local connected fractal dimensions and lacunarity analyses of 60 degrees fluorescein angiograms. *Investig. ophthalmology & visual science* **36**, 2749–2755 (1995).

18. Karperien, A. Fraclac for imagej. *Charles Sturt Univ.* (2013).

19. Virtanen, P. *et al.* SciPy 1.0: Fundamental Algorithms for Scientific Computing in Python. *Nat. Methods* **17**, 261–272, DOI: 10.1038/s41592-019-0686-2 (2020).

20. Stineman, R. W. A consistently well-behaved method of interpolation. *Creat. Comput.* **6**, 54–57 (1980).

21. Stephens, M. A. Use of the kolmogorov–smirnov, cramer–von mises and related statistics without extensive tables. *J. Royal Stat. Soc. Ser. B (Methodological)* **32**, 115–122 (1970).

22. MacEwan, D. M., Wilson, M., Brindley, G. & Brown, G. Interlayer and intercalation complexes of clay minerals. *Cryst. structures clay minerals their X-ray identification* **5**, 197–248 (1980).

23. Sherwood, T. K. The drying of solids—i. *Ind. & Eng. Chem.* **21**, 12–16 (1929).

24. Silling, S. A. & Askari, E. A meshfree method based on the peridynamic model of solid mechanics. *Comput. & structures* **83**, 1526–1535 (2005).

25. Trageser, J. & Seleson, P. Bond-based peridynamics: A tale of two poisson's ratios. *J. Peridynamics Nonlocal Model.* **2**, 278–288 (2020).

26. Silling, S. A. Reformulation of elasticity theory for discontinuities and long-range forces. *J. Mech. Phys. Solids* **48**, 175–209 (2000).

27. Jones, R. E., Rimsza, J. M., Trageser, J. E. & Hogancamp, J. R. Simulation of hardened cement degradation and estimation of uncertainty in predicted failure times with peridynamics. *Constr. Build. Mater.* **286**, 122927 (2021).

28. Otter, J. R. H., Cassell, A. C., Hobbs, R. E. & POISSON. Dynamic relaxation. *Proc. Inst. Civ. Eng.* **35**, 633–656 (1966).

29. Wood, W. L. Note on dynamic relaxation. *Int. J. for Numer. Methods Eng.* **3**, 145–147 (1971).

30. Kilic, B. & Madenci, E. An adaptive dynamic relaxation method for quasi-static simulations using the peridynamic theory. *Theor. Appl. Fract. Mech.* **53**, 194–204 (2010).

31. Justnes, H. *et al.* 13 chemical shrinkage of cement paste, mortar and concrete. In *Autogenous shrinkage of concrete: proceedings of the international workshop, organised by JCI (Japan Concrete Institute), Hiroshima, June 13-14, 1998*, 211 (Taylor & Francis, 1999).

32. Tazawa, E.-i., Miyazawa, S. & Kasai, T. Chemical shrinkage and autogenous shrinkage of hydrating cement paste. *Cem. concrete research* **25**, 288–292 (1995).

33. Dauti, D. *et al.* Modeling concrete exposed to high temperature: Impact of dehydration and retention curves on moisture migration. *Int. J. for Numer. Anal. Methods Geomech.* **42**, 1516–1530 (2018).

34. Alonso, C. & Fernandez, L. Dehydration and rehydration processes of cement paste exposed to high temperature environments. *J. materials science* **39**, 3015–3024 (2004).

35. Chen, J. J., Thomas, J. J. & Jennings, H. M. Decalcification shrinkage of cement paste. *Cem. concrete research* **36**, 801–809 (2006).

36. Zhang, Q. & Ye, G. Dehydration kinetics of portland cement paste at high temperature. *J. thermal analysis calorimetry* **110**, 153–158 (2012).

37. Kovler, K. & Zhutovsky, S. Overview and future trends of shrinkage research. *Mater. structures* **39**, 827–847 (2006).

38. Bala, P., Samantaray, B. & Srivastava, S. Dehydration transformation in ca-montmorillonite. *Bull. Mater. Sci.* **23**, 61–67 (2000).

39. Ebrahimi, D., Pellenq, R. J.-M. & Whittle, A. J. Nanoscale elastic properties of montmorillonite upon water adsorption. *Langmuir* **28**, 16855–16863 (2012).

40. Vanorio, T., Prasad, M. & Nur, A. Elastic properties of dry clay mineral aggregates, suspensions and sandstones. *Geophys. J. Int.* **155**, 319–326 (2003).

41. Wang, J.-J., Zhu, J.-G., Chiu, C. & Zhang, H. Experimental study on fracture toughness and tensile strength of a clay. *Eng. Geol.* **94**, 65–75 (2007).

42. Brigatti, M. F., Galan, E. & Theng, B. Structure and mineralogy of clay minerals. In *Developments in clay science*, vol. 5, 21–81 (Elsevier, 2013).

43. Annan, E. *et al.* Clay mixtures and the mechanical properties of microporous and nanoporous ceramic water filters. *J. Mater. Civ. Eng.* **28**, 04016105 (2016).

44. Bobaru, F. & Zhang, G. Why do cracks branch? a peridynamic investigation of dynamic brittle fracture. *Int. J. Fract.* **196**, 59–98 (2015).

45. Bobaru, F. & Hu, W. The meaning, selection, and use of the peridynamic horizon and its relation to crack branching in brittle materials. *Int. journal fracture* **176**, 215–222 (2012).
